# The USP7-TRIM27 axis mediates non-canonical PRC1.1 function and is a druggable target in leukemia

**DOI:** 10.1016/j.isci.2021.102435

**Published:** 2021-04-16

**Authors:** Henny Maat, Tjerk Jan Atsma, Shanna M. Hogeling, Aida Rodríguez López, Jennifer Jaques, Mirjam Olthuis, Marcel P. de Vries, Chantal Gravesteijn, Annet Z. Brouwers-Vos, Nisha van der Meer, Suzan Datema, Jonas Salzbrunn, Gerwin Huls, Roy Baas, Joost H.A. Martens, Vincent van den Boom, Jan Jacob Schuringa

**Affiliations:** 1 Department of Experimental Hematology, Cancer Research Center Groningen, University Medical Center Groningen, University of Groningen, Hanzeplein 1, 9713 GZ Groningen, The Netherlands; 2 Department of Pharmacy, Interfaculty Mass Spectrometry Center, University of Groningen, A. Deusinglaan 1, 9713 AV Groningen, The Netherlands; 3 Department of Pediatrics, Center for Liver, Digestive, and Metabolic Diseases, University Medical Center Groningen, University of Groningen, Hanzeplein 1, 9713 GZ Groningen, The Netherlands; 4 Division of Biochemistry and Oncode Institute, Netherlands Cancer Institute, 1066 CX Amsterdam, the Netherlands; 5 Department of Molecular Biology, RIMLS, Radboud University, Nijmegen, The Netherlands

**Keywords:** Molecular Biology, Cell Biology, Cancer

## Abstract

In an attempt to unravel functionality of the non-canonical PRC1.1 Polycomb complex in human leukemogenesis, we show that USP7 and TRIM27 are integral components of PRC1.1. USP7 interactome analyses show that PRC1.1 is the predominant Polycomb complex co-precipitating with USP7. USP7 inhibition results in PRC1.1 disassembly and loss of chromatin binding, coinciding with reduced H2AK119ub and H3K27ac levels and diminished gene transcription of active PRC1.1-controlled loci, whereas H2AK119ub marks are also lost at PRC1 loci. TRIM27 and USP7 are reciprocally required for incorporation into PRC1.1, and TRIM27 knockdown partially rescues USP7 inhibitor sensitivity. USP7 inhibitors effectively impair proliferation in AML cells *in vitro*, also independent of the USP7-MDM2-TP53 axis, and MLL-AF9-induced leukemia is delayed *in vivo* in human leukemia xenografts. We propose a model where USP7 counteracts TRIM27 E3 ligase activity, thereby maintaining PRC1.1 integrity and function. Moreover, USP7 inhibition may be a promising new strategy to treat AML patients.

## Highlights

• We identify USP7 and TRIM27 as integral components of non-canonical PRC1.1
• USP7 inhibition results in PRC1.1 disassembly and loss of chromatin binding
• TRIM27 and USP7 are reciprocally required for incorporation into PRC1.1
• USP7 inhibitors effectively impair AML proliferation, also independent of TP53

## Graphical abstract

**Figure:**
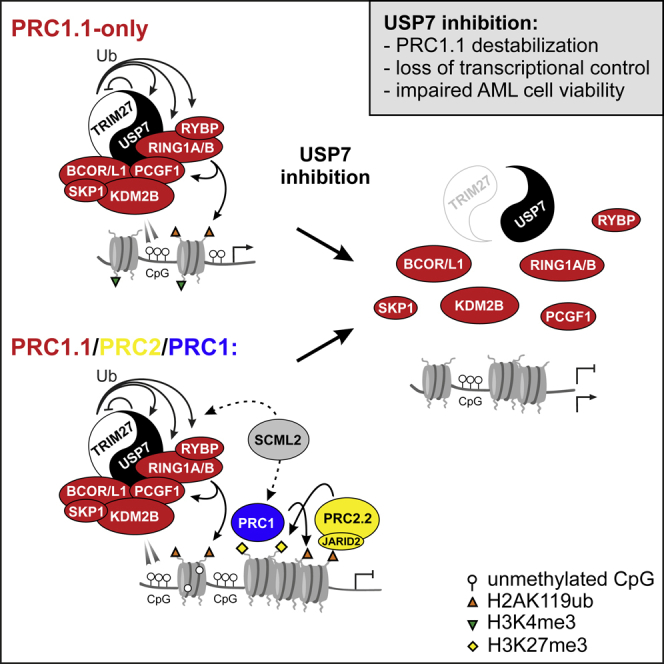

## Introduction

Patients with acute myeloid leukemia (AML) often have a poor prognosis despite treatment with intensive chemotherapy and allogeneic stem cell transplantation. Dependent on risk category, overall survival for adult AML patients varies between 10% and 60% (Arber et al., 2016; Burnett et al., 2011). AML mostly affects the elderly, as 75% of the cases occur in patients >60 years of age, who have a particularly poor outcome (Klepin, 2016; Rucker et al., 2012). These older patients usually have karyotypes associated with unfavorable risk and also TP53 mutations, and do not respond well to standard chemotherapy (Bowen et al., 2009; Hou et al., 2015; Rucker et al., 2012). Therefore, alternative therapies need to be developed to achieve more effective treatment of AML patients, particularly in unfavorable subtypes.

A recurrent challenge in AML treatment is the notion that standard-of-care chemotherapeutic approaches do not effectively target quiescent leukemic stem cell (LSC) populations, and as a consequence relapse of disease occurs frequently. A thorough understanding of how LSCs self-renew and maintain their quiescent state is therefore essential to improve AML treatment. We and others have shown that Polycomb group (PcG) proteins are important regulators of normal hematopoietic stem cell (HSC) and LSC fate (Iwama et al., 2004; Lessard and Sauvageau, 2003; Rizo et al., 2008; Rizo et al., 2010; Rizo et al., 2009; van den Boom et al., 2016; van den Boom et al., 2013; Yuan et al., 2011). This family of epigenetic regulators controls both stem cell self-renewal and cell lineage specification by regulating gene transcription through histone modifications and chromatin remodeling (Simon and Kingston, 2013).

PcG proteins form multi-protein chromatin-modifying complexes of which the canonical Polycomb repressive complex 1 (PRC1) and 2 (PRC2) were first identified (Simon and Kingston, 2013). PRC1 and PRC2 can deposit H2AK119ub and H3K27me3 marks, respectively, and can be independently tethered to, and silence, shared Polycomb target genes. All PRC1 subunits have multiple paralogs, suggesting that various specific PRC1 complexes can coexist in the cell. Indeed in various cell types including hematopoietic stem/progenitor cells and mES cells, a lack of redundancy between PRC1 subunit paralogs was observed, and complex composition can change upon lineage specification (Klauke et al., 2013; Morey et al., 2012; Morey et al., 2015; O'Loghlen et al., 2012; van den Boom et al., 2013).

In addition to canonical PRC1, either containing BMI1/PCGF4 (PRC1.4) or MEL18/PCGF2 (PRC1.2), various non-canonical PRC1 complexes have been identified, including PRC1.1, PRC1.3/1.5, and PRC1.6 (Gao et al., 2012; Tavares et al., 2012; van den Boom et al., 2013). We previously identified an essential role for the PRC1.1 complex in human leukemias (van den Boom et al., 2016). A potential oncogenic role of PRC1.1 is underlined by the fact that KDM2B is overexpressed in leukemias, breast cancer, and pancreatic cancer and conversely knockdown of KDM2B abrogated tumorigenicity (Andricovich et al., 2016; He et al., 2011; Kottakis et al., 2014; Ueda et al., 2015; van den Boom et al., 2016). PRC1.1 was first identified by the purification of the BCOR protein, which was found to interact with RING1A/B, RYBP, PCGF1, SKP1, and KDM2B (Gearhart et al., 2006; Sanchez et al., 2007). We and others have found that PRC1.1 is targeted to genes together with PRC1.2/1.4 and PRC2, where these complexes likely cooperatively silence gene expression (Farcas et al., 2012; He et al., 2013; van den Boom et al., 2016; Wu et al., 2013). However, in addition, we also observed a large group of active genes that were targeted by PRC1.1, in the absence of PRC2, suggesting that PRC1.1 function is not restricted to silenced genes (van den Boom et al., 2016).

Here, using LC-MS/MS-based interactome studies, we set out to characterize the PRC1.1 complex in detail and explore its targetability in AML. Thus, we identify two novel subunits of PRC1.1, the ubiquitin-specific peptidase (USP)7 and the E3 ligase tripartite motif (TRIM)27. USP7 and TRIM27 are known to interact and play a role in various cellular processes including TNFα-induced apoptosis (Zaman et al., 2013), endosomal protein recycling (Hao et al., 2015), and antiviral type I interferon signaling (Cai et al., 2018). USP7 function has previously also been linked to canonical PRC1 function. USP7 was found to interact with SCML2 (Lecona et al., 2015; Luo et al., 2015), a subunit of PRC1, and other work showed that USP7 is required for PRC1-mediated repression of target genes and regulated ubiquitination status of BMI1/PCGF4 and MEL18/PCGF2 (Maertens et al., 2010).

Protein ubiquitination is an important post-translational modification that controls the stability of almost all cellular proteins. Mono-ubiquitination impacts the activity of proteins or can promote or prevent protein-protein interactions, whereas poly-ubiquitinated proteins are typically targeted to and degraded by the proteasome (Hershko, 1983). USP7 is a ubiquitin-specific protease that displays a wide range of activities (Kim and Sixma, 2017), making it an attractive candidate target for cancer treatment. USP7 inhibition destabilizes MDM2, resulting in increased levels of TP53, and recently a number of USP7-specific inhibitors were generated that effectively targeted various human cancer cells presumably in a TP53-dependent manner (Gavory et al., 2018; Kategaya et al., 2017; Turnbull et al., 2017). However, TP53-independent roles exist as well (Bhattacharya et al., 2018; Nicholson and Suresh Kumar, 2011).

Our data described here indicate that USP7 and TRIM27 are integral components of the PRC1.1 complex. Furthermore, inhibition of USP7 results in PRC1.1 complex disassembly and dissociation from chromatin, with concomitant changes in the epigenetic state and gene expression of target loci. We show that USP7 activity is essential for leukemic cells and targeting of USP7 might provide an alternative therapeutic approach for leukemia, also for the most aggressive subtypes of AML that harbor mutations in TP53.

## Results

### Identification of USP7 and TRIM27 as PRC1.1 subunits

**Figure 1:**
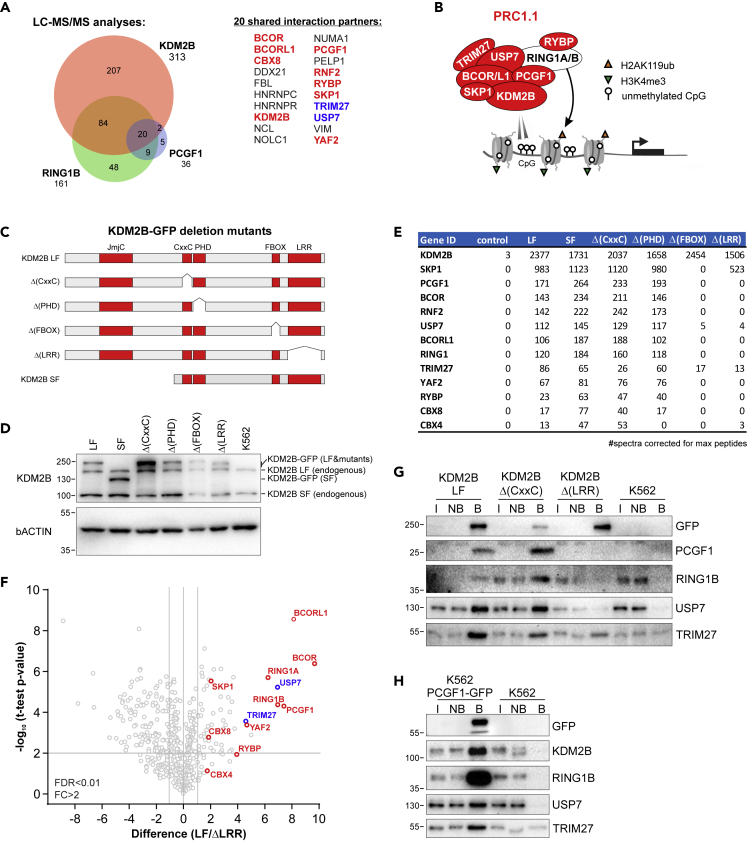
LC-MS/MS-based identification of new PRC1.1 subunits (A) LC-MS/MS analysis of KDM2B-GFP, GFP-RING1B, and PCGF1-GFP pull outs in K562 cells showing 20 overlapping interaction partners including PRC1.1 subunits (in red) and new interaction partners such as USP7 and TRIM27 (in blue). (B) Schematic overview of PRC1.1 subunit composition. PRC1.1 preferentially binds to non-methylated CpG islands via the CxxC domain of KDM2B. The ubiquitination of histone H2A on lysine 119 is mediated by RING1A/B. (C) Schematic representation of KDM2B deletion mutants and the endogenously expressed long form (LF) and short form (SF; lacking the JmjC domain) of KDM2B. (D) Western blots showing expression of GFP-fusions at near to endogenous KDM2B expression levels. (E) Table showing LC-MS/MS data of PRC1.1 subunits and other PcG proteins co-precipitating with wild-type and mutant forms of KDM2B-GFP. Numbers indicate the average total spectra counts, as measured in triplicate, corrected for maximally identifiable peptides based on in silico protein digests. (F) Volcano plot showing MaxQuant-based label-free quantification of triplicate measurements of KDM2B LF-GFP and KDM2B Δ(LRR)-GFP pull outs. PRC1.1 subunits are indicated in red and USP7 and TRIM27 in blue. (G) Western blot analysis of independent pull outs of (mutant) KDM2B-GFP with input (I, 1/90^th^), non-bound (NB, 1/90^th^), and bound (B, 1/3^rd^) fractions loaded and stained with antibodies against GFP, PCGF1, RING1B, USP7, and TRIM27. (H) PCGF1-GFP pull outs with input (I, 1/90^th^), non-bound (NB, 1/90^th^), and bound (B, 1/3^rd^) and stained with antibodies directed against GFP, KDM2B, RING1B, USP7, and TRIM27. See also Figure S1 and Tables S1, S2, and S3.

Previously, we identified non-canonical PRC1.1 as a critically important Polycomb complex for the survival of human leukemic stem cells (van den Boom et al., 2016). To better understand the function of PRC1.1 and explore its targetability in leukemic cells, we set out to further study the PRC1.1 interactome in detail in leukemic cells. Nuclear extracts were prepared from human K562 cells stably expressing KDM2B-GFP, GFP-RING1B, PCGF1-GFP, or non-transduced control cells, followed by anti-GFP pull outs and LC-MS/MS analysis to identify interacting proteins. We identified 313 KDM2B-GFP interaction partners, 161 GFP-RING1B interacting proteins, and 36 proteins co-precipitating with PCGF1-GFP, of which 20 proteins overlapped between these three groups (Figure 1A, Table S1). The overlapping fraction contained all known PRC1.1 subunits (Figures 1A and 1B).

Within this fraction, we also identified the deubiquitinase USP7 and the E3 ligase TRIM27. To investigate how USP7 and TRIM27 interact with KDM2B-GFP, we stably expressed wild-type KDM2B (long-form; LF) and various KDM2B deletion mutants lacking the CxxC, PHD, FBOX, or LRR domain in K562 cells (Figure 1C). In addition, we stably expressed a naturally occuring short form (SF) of KDM2B in which the JmjC domain is absent. All fusion proteins were expressed at near to endogenous expression levels (Figure 1D). LC-MS/MS analyses of KDM2B-GFP pull outs showed that the maximal number of spectral counts of co-precipitating PRC1.1 subunits was strongly reduced when either the FBOX or LRR domain was deleted (Figure 1E, Table S2). Precipitation of PRC1.1 subunits bound to KDM2B was maintained in the CxxC mutant, suggesting that PRC1.1 complex does not require association with chromatin. Importantly, USP7 and TRIM27 co-precipitation with KDM2BΔ(FBOX) and KDM2BΔ(LRR) was also strongly reduced compared with wild-type KDM2B (Figure 1E). Label-free quantification of LC-MS/MS data of KDM2B LF-GFP and KDM2BΔ(LRR)-GFP pull outs showed a significant reduction of the interaction of KDM2BΔ(LRR)-GFP with all PRC1.1 subunits, including USP7 and TRIM27 (Figure 1F, Table S3). Of note, SKP1 binding to KDM2B was only sensitive to deletion of the FBOX domain. Our data are in line with earlier work showing that KDM2B integration into PRC1.1 depends on the leucine-rich repeat region in the C-terminus of KDM2B (Wong et al., 2016; Wu et al., 2013). LRR dependency of the KDM2B interaction with PCGF1, RING1B, USP7, and TRIM27 was confirmed by western blot analyses of independent pull outs (Figure 1G). Interestingly, KDM2BΔ(LRR)-GFP pull outs still showed some residual USP7/TRIM27 binding, suggesting that USP7/TRIM27 incorporation into PRC1.1 is likely dependent on various PRC1.1 subunits, including RING1B, which was previously shown to interact with USP7 (de Bie et al., 2010) and KDM2B. We also performed independent pull outs to validate the interaction of USP7 and TRIM27 with PCGF1-GFP (Figure 1H).

Apart from mutual KDM2B-GFP, GFP-RING1B, and PCGF1-GFP interaction partners, we identified proteins that specifically interacted with one of these proteins (Figure 1A). GFP-RING1B-specific interaction partners (48) included various subunits of canonical (PRC1.2/1.4) and other variant PRC1 complexes (PRC1.3/1.5/1.6), and RING1A specifically co-precipitated with PCGF1-GFP and KDM2B-GFP but not with GFP-RING1B. We also identified 207 proteins that specifically interacted with KDM2B. Gene ontology analyses revealed a strong association with processes such as gene expression, RNA processing, RNA splicing, transcription from RNAPII promoters, and histone ubiquitination (Figure S1A). These interaction partners included various transcription regulatory complexes including the super elongator complex (SEC) comprised of pTEF-b and MLL-fusion partners/ELL-associated proteins, FACT, the PAF complex, and Mediator complex, underlining that KDM2B also interacts with complexes that regulate RNAPII transcription initiation, pause-release, and transition into processive elongation (Figure S1B). Interestingly, these KDM2B-specific interactions with transcription regulatory complexes showed distinct KDM2B domain dependencies. Where most interacting complexes were dependent on the CxxC domain (i.e. pTEFb, PAF complexes), the interaction with the Mediator complex was lost upon deletion of the FBOX/LRR domain (Figure S1B).

### PRC1.1 is the predominant Polycomb complex interacting with USP7

**Figure 2:**
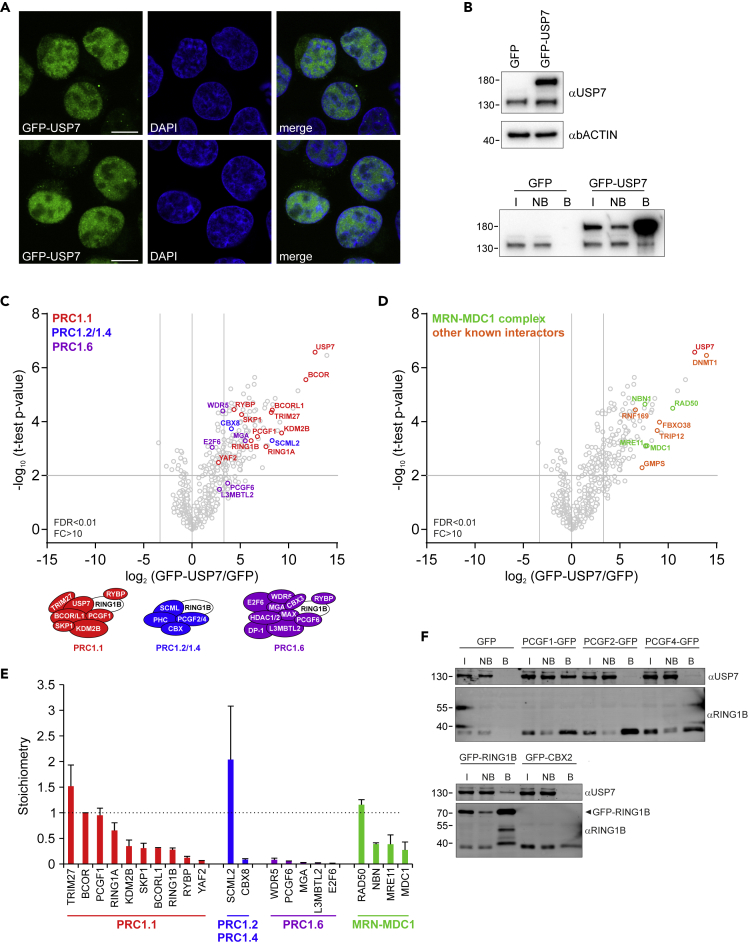
GFP-USP7 predominantly interacts with PRC1.1 (A) Confocal images of fixed K562 GFP-USP7 cells stained with DAPI. Scale bars represent 25 μm. (B) Western blots showing relative expression of GFP-USP7 versus endogenous USP7 in K562 cells and efficient precipitation of GFP-USP7 using GFP-Trap beads. Input (I, 1/90^th^), non-bound (NB, 1/90^th^), and bound (B, 1/3^rd^) are indicated. (C) Volcano plot showing enrichment of GFP-USP7-specific interaction partners in GFP and GFP-USP7 pull outs. Pull outs were performed in triplicate on K562 GFP and GFP-USP7 cells, and samples were analyzed using LC-MS/MS, followed by data analysis using MaxQuant and Perseus software. Statistical analysis was performed using Student's t test (false discovery rate (FDR) < 0.01; fold change (FC) > 10). PRC1.1, PRC1.2/1.4, and PRC1.6 subunits are indicated in red, blue, and purple respectively. (D) Volcano plot showing enrichment of previously identified USP7 interaction partners (orange), including the MRN-MDC1 complex (green). Statistical analysis was performed using Student's t test (false discovery rate (FDR) < 0.01; fold change (FC) > 10). (E) Intensity-based absolute quantification (iBAQ) value-based calculation of relative stoichiometry values relative to BCOR. (F) Western blot analysis of independent pull outs of GFP, PCGF1-GFP, PCGF2-GFP, PCGF4-GFP, GFP-RING1B, and GFP-CBX2 where input (I, 1/90^th^), non-bound (NB, 1/90^th^), and bound (B1/3^rd^) fractions are loaded and stained with USP7 and RING1B antibodies. See also Table S4.

USP7 is a protein involved in various cellular processes and possesses DUB activity toward numerous substrates (Kim and Sixma, 2017). To further validate the USP7-PRC1.1 interaction we performed the reverse experiment by means of USP7 pull outs. We lentivirally expressed a GFP-TEV-FLAG-3C-USP7 fusion (previously generated by the T.K. Sixma lab; hereafter GFP-USP7) in K562 cells in order to perform pull outs and interactome studies. Importantly, GFP was fused to the amino-terminus of USP7 because the carboxy-terminal Ubl domains of USP7 fold back on the catalytic domain and are important to boost the low intrinsic catalytic activity of USP7 (Faesen et al., 2011; Kim et al., 2019). Confocal imaging of GFP-USP7 cells revealed a diffuse nuclear localization but also some cytoplasmic localization (Figure 2A). GFP-USP7 was slightly overexpressed compared with endogenous USP7 protein expression and could be efficiently co-precipitated using GFP-Trap beads (Figure 2B).

Next, we performed label-free quantification of pull outs on K562 cells expressing either GFP or GFP-USP7. All PRC1.1 subunits were significantly and strongly enriched in GFP-USP7 pull outs, and PRC1.1 subunits (i.e. BCOR, KDM2B) were among the top interactors with GFP-USP7 (Figure 2C, Table S4). Previous work has shown that USP7 also interacts with the canonical PRC1 subunit SCML2, which may form a bridge toward PRC1 (Lecona et al., 2015; Luo et al., 2015). We also observed a strong interaction with SCML2, but except for CBX8 we did not find other canonical PRC1 (PRC1.2/1.4) subunits to co-precipitate with GFP-USP7. In addition, we did observe a weak enrichment of various subunits of the non-canonical PRC1.6 complex, suggesting that USP7 may interact with this variant PRC1 complex as well (Figure 2C). We also identified previously described USP7 interaction partners, including DNMT1 (Cheng et al., 2015), GMPS (van der Knaap et al., 2005), FBXO38 (Georges et al., 2019), TRIP12 (Liu et al., 2016), and the MRN-MDC1 complex (Su et al., 2018) (Figure 2D). Next, we used the intensity-based absolute quantification (iBAQ) values to calculate the relative stoichiometry of individual subunits of the identified PRC1 complexes relative to BCOR, which was the most abundant PRC1.1 subunit detected in GFP-USP7 pull outs (Figure 2E). Across all PRC1.1 subunits, PCGF1 and RING1A showed a stoichiometry comparable to BCOR. Lower stoichiometries of BCORL1 and RING1B may be explained based on their mutual exclusiveness, and potentially competition, with BCOR and RING1A for incorporation into PRC1.1. Interestingly, the heterodimer KDM2B-SKP1 was co-precipitated with slightly lower stoichiometries, indicating that not all GFP-USP7-precipitated PRC1.1 complexes contain KDM2B-SKP1. This observation is in line with a previously proposed hierarchical assembly model for PRC1.1 where BCOR/BCORL1 and PCGF1 can form a subcomplex that can exist on its own and independently interacts with a KDM2B/SKP1 heterodimer (Wong et al., 2016). RYBP and YAF2, which also interact with PRC1.1 in a mutually exclusive manner, were incorporated at substoichiometric levels. Compared with PRC1.1, the relative abundance of PRC1.6 subunits was much lower but comparable among each other. Also subunits of the MRN/MDC1 complex were co-precipitated with comparable stoichiometries. Importantly, the PRC1 subunits SCML2 and CBX8 showed very distinct stoichiometries, suggesting that a large fraction of SCML2 is not interacting with CBX8. Independent pull outs of PCGF1-GFP, PCGF2-GFP, PCGF4-GFP, GFP-RING1B, and GFP-CBX2, and independent LC-MS/MS analyses (van den Boom et al., 2016), only showed strong USP7 co-precipitation with PCGF1-GFP and GFP-RING1B (Figure 2F). These data suggest that the interaction between USP7 and SCML2 may take place outside the PRC1 complex or that SCML2 acts as bridging factor between USP7 and PRC1. Taken together, our data show that PRC1.1 is the predominant Polycomb complex co-precipitating with GPF-USP7 in leukemic cells.

### USP7 inhibition results in disassembly of the PRC1.1 complex

**Figure 3:**
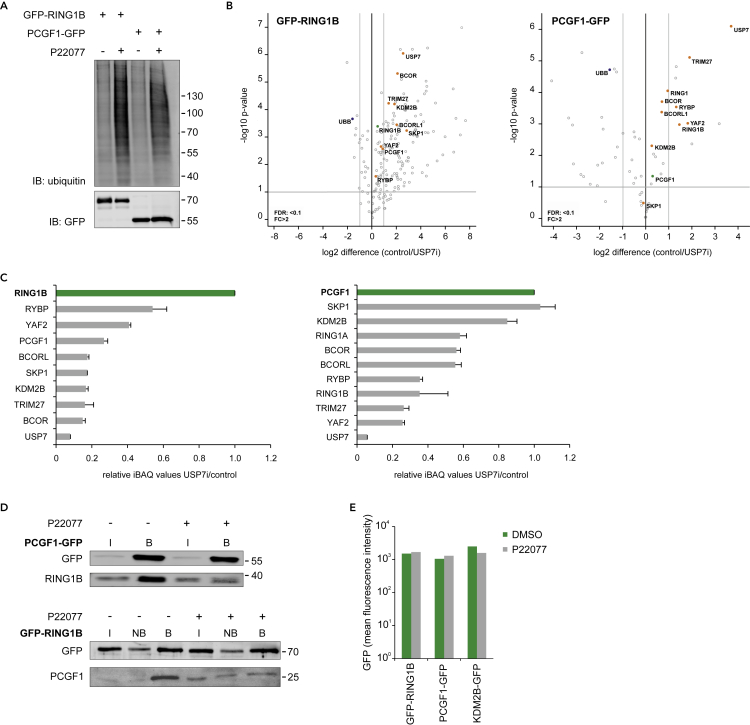
USP7 deubiquitinase activity is essential for PRC1.1 integrity (A) Purification of His-tagged ubiquitinated proteins under denaturing conditions in GFP-RING1B and PCGF1-GFP cells treated with DMSO or P22077 for 24 h (30 μM) followed by western blot analysis. (B) Volcano plot showing differential interaction of GFP-RING1B (left) and PCGF1-GFP (right; both indicated in green) with PRC1.1 subunits (highlighted in orange) as measured by label-free quantification (LFQ) of LC-MS/MS data in triplicate. The UBB protein is indicated in blue. Statistical analysis was performed using Student's t test (false discovery rate (FDR) < 0.1; fold change (FC) > 2). (C) Intensity-based absolute quantification (iBAQ) value ratios of several identified PRC1.1 proteins as identified in GFP-RING1B (left) and PCGF1-GFP (right) pull outs from P22077- or DMSO-treated cells. Data are shown as mean ± SD (n = 2 or 3). (D) Western blot of GFP pull outs on PCGF1-GFP and GFP-RING1B in the absence (−) or presence (+) of P22077 (72 h, 30 μM) probed with antibodies for GFP, RING1B, and PCGF1. Input (I, 1/90^th^), non-bound (NB, 1/90^th^), and bound (B, 1/3^rd^) fractions are shown. (E) Mean fluorescent intensity (MFI) analysis of K562 cells expressing either GFP-RING1B, PCGF1-GFP, or KDM2B-GFP treated with DMSO or P22077 for 72 h. See also Table S5.

Next, we investigated the molecular role of USP7 within PRC1.1. Deubiquitinating enzymes (DUBs) exert a variety of important cellular functions, including the control over protein stabilization or degradation, protein localization and protein activity or by modulating protein-protein interactions (Leznicki and Kulathu, 2017). We therefore questioned whether inhibition of the deubiquitinase USP7 might affect PRC1.1 stability and function. In general, DUB inhibitors increase overall protein polyubiquitination of many target proteins (Altun et al., 2011). Indeed, we observed an increase of polyubiquitinated proteins in K562 cells expressing GFP-RING1B or PCGF1-GFP treated for 24 h with the USP7 inhibitor P22077 (Figure 3A). Next, we investigated the effect of USP7 inhibition on the stability of PRC1.1. GFP pull outs were performed on nuclear extracts from K562 PCGF1-GFP and GFP-RING1B cells treated with DMSO or P22077 followed by LC/MS-MS analysis (Figure 3B and Table S5). Label-free quantification of LC-MS/MS data showed that interactions with several PRC1.1 proteins, highlighted in orange, were significantly reduced in both GFP-RING1B and PCGF1-GFP pull outs as a consequence of USP7 inhibition. The ubiquitin protein, UBB, was enriched in both PCGF1-GFP and GFP-RING1B pull outs upon P22077 treatment, suggesting that these proteins or other Polycomb interaction partners were more ubiquitinated upon USP7 inhibition. Calculation of normalized iBAQ value ratios (P22077/DMSO) allowed us to investigate changes in PRC1.1 stoichiometry upon USP7 inhibition. Clearly, these data demonstrated a reduced interaction of PRC1.1 proteins with RING1B and PCGF1 after P22077 treatment (Figure 3C). Independent GFP pull outs performed on PCGF1-GFP and GFP-RING1B cell lines further confirmed that USP7 inhibition indeed resulted in reduced interaction with endogenous RING1B and PCGF1, respectively (Figure 3D). Importantly, input samples did not reveal reduced expression of GFP-RING1B, PCGF1-GFP, or KDM2B-GFP, which was further validated by FACS analysis (Figure 3E). Taken together, these data indicate that USP7 is essential for PRC1.1 complex integrity.

As our proteomics studies were conducted with exogenously expressed tagged proteins, it will be important to further substantiate these findings in experiments using endogenous tagging approaches. Currently we are employing CRISPR-Cas9 prime editing to tag endogenous proteins, and we are generating conditional knockout models using auxin-inducible degron (AID) tagging. Also, in our future studies we will aim at deciphering the exact ubiquitination sites within PRC1.1 proteins using a diGly proteomics approach (Kim et al., 2011; van der Wal et al., 2018; Xu et al., 2010) and determine how these are affected by USP7 inhibition.

### PRC1.1 chromatin binding relies on USP7 deubiquitinase activity

**Figure 4:**
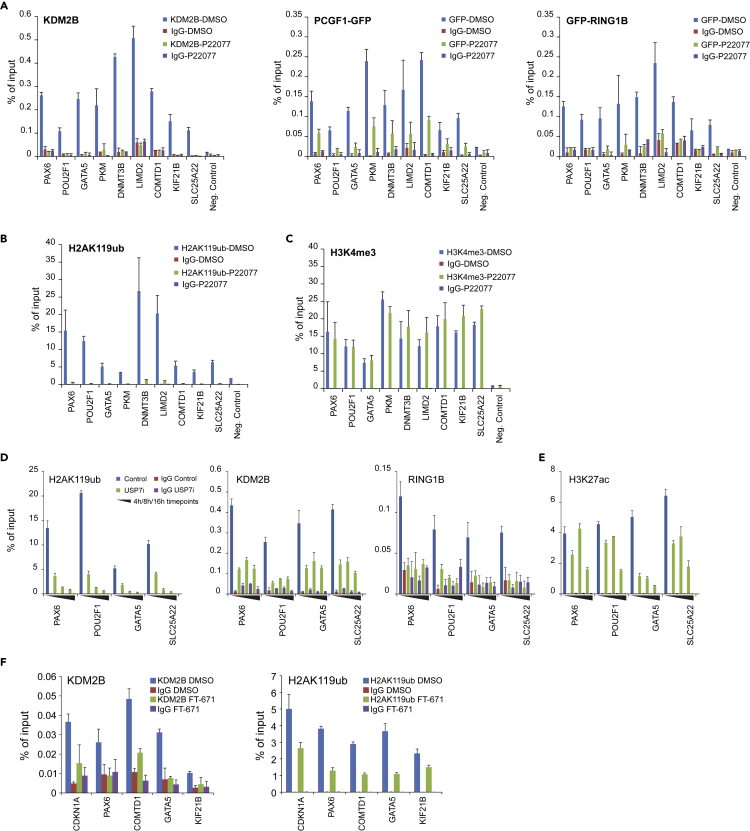
USP7 inhibition induces loss of PRC1.1 occupancy and H2AK119ub at target loci (A) ChIP-qPCRs on K562, K562 GFP-RING1B, and K562 PCGF1-GFP cells, treated with DMSO or P22077 (72 h, 30 μM), using antibodies against KDM2B or GFP. (B and C) ChIP-qPCRs on K562 cells using antibodies directed against H2AK119ub (B), H3K4me3 (C), on several PRC1.1 loci. Error bars represent SD of technical qPCR replicates. (D and E) ChIP-qPCRs on DMSO- or P22077-treated K562 cells for 4 h, 8 h, and 16 h using antibodies against H2AK119ub (D), GFP (reading out KDM2B-GFP and GFP-RING1B), and H3K27ac (E) on various PRC1.1 loci. Error bars represent SD of technical qPCR replicates. (F) ChIP-qPCRs on K562 cells treated with either DMSO or FT671 (48 h, 10 μM) using antibodies against KDM2B and H2AK119ub. Error bars represent SD of three biological replicate experiments. See also Figure S2.

To determine whether USP7-inhibition-induced disassembly of the PRC1.1 complex also affect PRC1.1 chromatin binding we performed chromatin immunoprecipitation (ChIP) experiments. Previously, we performed ChIP-seq on PCGF1/2/4, CBX2, RING1A/1B, KDM2B, H2AK119ub, and H3K27me3 to identify PRC1.1 target genes in K562 cells (van den Boom et al., 2016). Clearly, inhibition of USP7 resulted in a complete loss of endogenous KDM2B chromatin binding and strong reductions in PCGF1-GFP and GFP-RING1B interactions with PRC1.1 target genes (Figure 4A). Because RING1B mediates H2AK119 ubiquitination we hypothesized that USP7 inhibition may result in decreased H2AK119ub levels. Indeed, H2AK119ub was lost from PRC1.1 target loci (Figure 4B), indicating that USP7 inhibition has severe impact on PRC1.1 chromatin binding and as a consequence on H2AK119ub levels. Because the analyzed PRC1.1 loci are active loci in leukemic cells (van den Boom et al., 2016), we also investigated the levels of H3K4me3, but no changes were observed for this histone mark upon USP7 inhibition, highlighting that not all histone modifications are affected as a consequence of treatment (Figure 4C). To further investigate the kinetics of USP7-inhibition-induced loss of H2AK119ub, cells were cross-linked after 4 h, 8 h, and 16 h of treatment with P22077, followed by a ChIP for H2AK119ub, KDM2B-GFP, or GFP-RING1B (Figure 4D). The kinetics of H2AK119ub loss strongly correlated with reduced KDM2B-GFP or GFP-RING1B binding, suggesting that loss of de novo ubiquitination underlies these observations. In addition, H3K27ac levels were reduced upon USP7 inhibition, indicative for reduced transcriptional activity at these loci (Figure 4E). We validated our findings using FT671, a novel and more specific USP7 inhibitor (Turnbull et al., 2017), which also induced a clear loss of KDM2B binding and reduced H2AK119ub levels at PRC1.1 loci (Figure 4F).

The loss in H2AK119ub marks upon USP7-inhibition-resembled phenotypes observed with inhibition of the proteasome (Dantuma et al., 2006; Tavares et al., 2012). In addition, we and others have previously shown that exposure of cells to heat stress also results in a quick loss of H2AK119ub marks (Azkanaz et al., 2019; Mimnaugh et al., 1997), likely as a consequence of massive unfolding of proteins, of which a large fraction needs to be degraded by the proteasome. To investigate whether FT671-induced loss of H2AK119ub may be a consequence of proteasome inhibition, we compared the kinetics of loss of H2AK119ub upon treatment with the proteasome inhibitor MG132 and the USP7 inhibitor FT671. Although indeed treatment with both compounds resulted in loss of H2AK119ub, the kinetics were rather different, with MG132 already resulting in a decrease within 2 h of treatment, whereas the effects of FT671 became apparent at much later timepoints (Figure S2). This would argue that different molecular mechanisms are involved.

**Figure 5:**
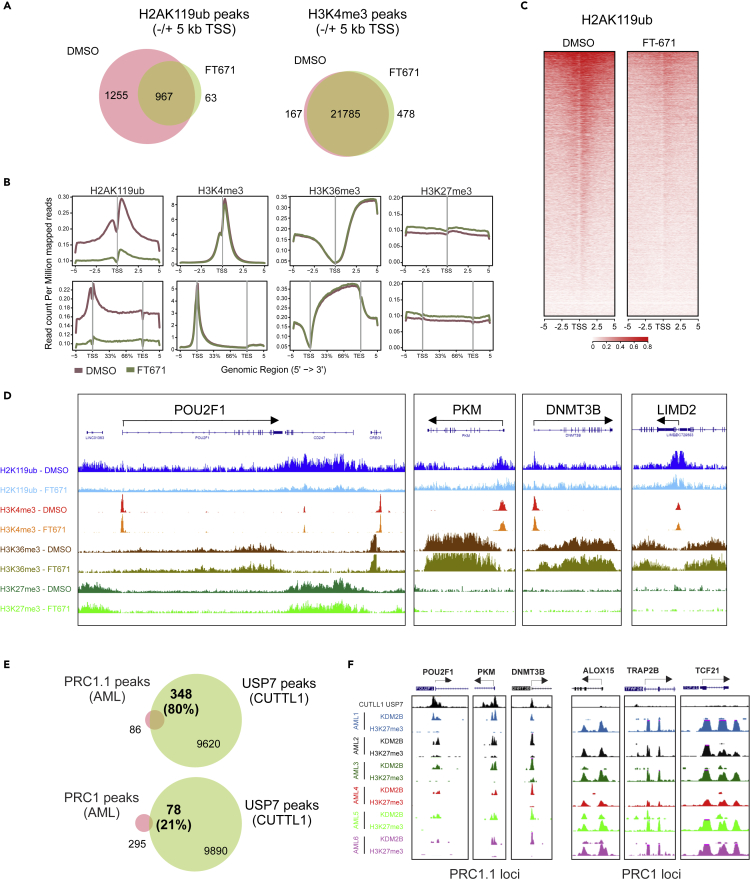
Genome-wide loss of H2AK119ub upon USP7 inhibition (A–D) ChIP-seq on K562 cells treated with either DMSO or FT671 (24 h, 10 μM) using antibodies against H2AK119ub, H3K4me3, H3K36me3, or H3K27me3. (A) Venn diagram depicting overlapping peaks −/+ 5kb from the transcription start site (TSS). (B) Density plots displaying epimarks around the TSS or across the gene body until + 5kb after the transcription end site (TES). (C) Heat maps of the H2AK119ub signal around the TSS. (D) Representative screenshots of epimarks at four PRC1.1 loci. (E) Non-canonical PRC1.1 or canonical PRC1 peaks shared between six primary AML CD34^+^ patient samples (van den Boom et al., 2016) were overlaid with USP7 peaks from CUTTL1 cells (Jin et al., 2019). (F) Representative screens shots of PRC1.1 and PRC1 loci depicting USP7 and KDM2B binding and H3K27me3 levels. See also Figure S3.

**Figure 6:**
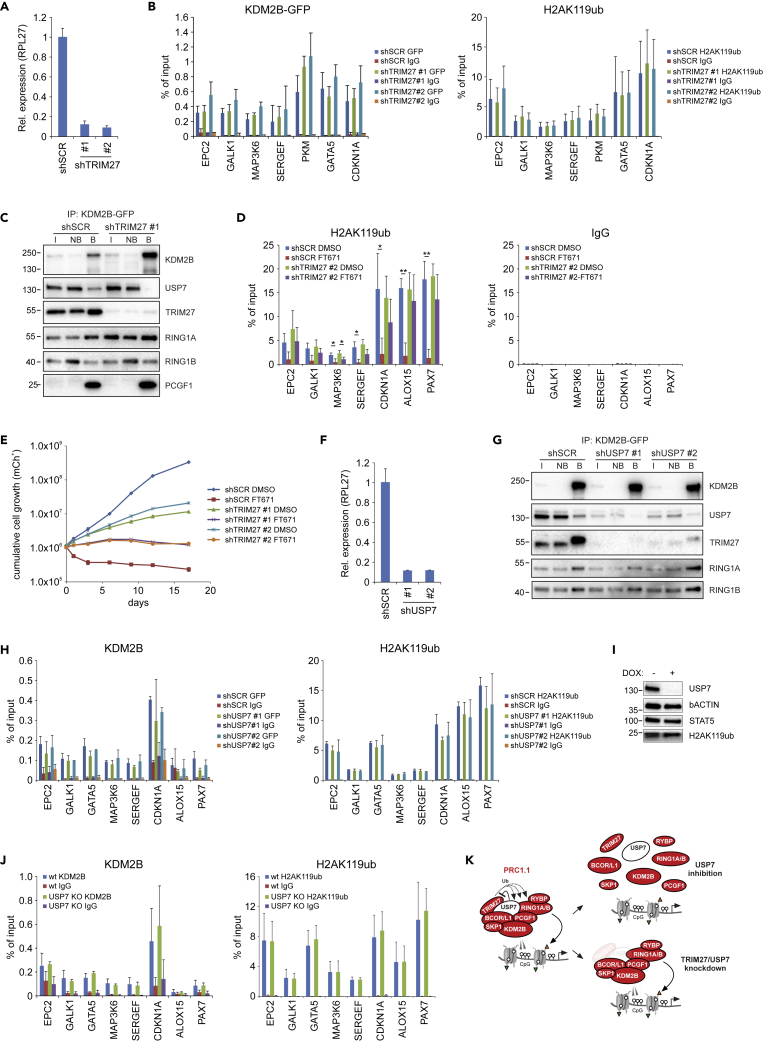
Loss of TRIM27 partially rescues USP7 inhibitor sensitivity (A) Knockdown efficiencies of two independent TRIM27 shRNAs in K562 cells. Error bars represent SD from technical triplicates. (B) KDM2B-GFP and H2AK119ub ChIP-qPCRs with error bars representing SD based on three independent experiments, statistical analysis was performed using Student's t test; ∗p < 0.05 and ∗∗p < 0.01. (C) Western blot analysis of KDM2B-GFP pull outs on K562 cells expressing SCR or TRIM27 (#1) shRNAs using the indicated antibodies. Input (I, 1/90^th^), non-bound (NB, 1/90^th^), and bound (B, 1/3^rd^) as indicated. (D) H2AK119ub ChIP-qPCR on K562 cells expressing SCR or TRIM27 (#2) shRNAs and treated with DMSO or FT671 (48 h, 10μM). Error bars represent SD based on three independent experiments; statistical analysis was performed using Student's t test; ∗p < 0.05 and ∗∗p < 0.01. (E) Cumulative cell proliferation of K562 cells expressing SCR or TRIM27 (#1 or #2) shRNAs and treated with DMSO or FT671. Error bars represent SD based on two independent experiments. (F) Knockdown efficiencies of two independent USP7 shRNAs in K562 cells. Error bars represent SD from technical triplicates. (G) Western blot analysis of KDM2B-GFP pull outs on K562 cells expressing SCR or USP7 (#1 and #2) shRNAs using the indicated antibodies. Input (I, 1/90^th^), non-bound (NB, 1/90^th^), and bound (B, 1/3^rd^) as indicated. (H) Endogenous KDM2B and H2AK119ub ChIP-qPCRs on K562 cells expressing SCR of USP7 (#1 and #2) shRNAs. Error bars represent SD based on three independent experiments; statistical analysis was performed using Student's t test; ∗p < 0.05 and ∗∗p < 0.01. (I) Western blot analysis of THP-1 doxycycline-inducible CRISPR/Cas9 USP7 knockout cells treated with doxycycline for 3 days and subsequent culture for 4 days and stained with the indicated antibodies. (J) Endogenous KDM2B and H2AK119ub ChIP-qPCRs on doxycycline-treated cells (day 6). Error bars represent SD based on three independent experiments; statistical analysis was performed using Student's t test; ∗p < 0.05 and ∗∗p < 0.01. (K) Graphical abstract of effects of USP7 inhibition versus USP7 or TRIM27 knockdown. See also Figure S4.

In order to obtain a genome-wide view on the effect of USP7 inhibition on histone modifications ChIP-seq was performed. These data showed a strong decrease in identified H2AK119ub peaks upon FT671 treatment (Figure 5A). The H2AK119ub signal at enriched transcription start sites and through gene bodies was also strongly reduced (Figures 5B and 5C), in line with our ChIP-qPCR data. In contrast, genome-wide H3K4me3 marks were only marginally affected, whereas H3K36me3 marks were slightly increased upon USP7 inhibition (Figures 5A and 5B). We also observed a slight increase in H3K27me3 marks (Figure 5B). Genome-wide effects of USP7 inhibition on H3K27ac marks will be investigated in future studies. When specifically investigating previously annotated genes occupied by PRC1.1, PRC1, and loci occupied by both PRC1.1 and PRC1 (van den Boom et al., 2016), we observed that the loss of H2AK119ub was observed in all three gene categories but was most significant at PRC1.1 loci (Figure S3). These data indicate that, even though we failed to detect strong direct interactions between USP7 and canonical PRC1, the effects of USP7 inhibition on H2AK119ub levels are clearly detected at PRC1-specific loci as well, possibly as a consequence of the USP7-SCML2 interaction bridging toward PRC1. In line, we also observed that the interaction between SCML2 and RING1B was lost upon USP7 inhibition (Table S5). Also in ChIP-qPCR studies on canonical PRC1 loci (PAX7 and ALOX15) and loci classified as “both” that are bound by both canonical PRC1 and non-canonical PRC1.1 (CDKN1A), we observed that inhibition of USP7 enzymatic activity resulted in reduced H2AK119ub levels (Figure 6D). These data indicate that the effects of USP7 inhibition on the epigenome and consequently the transcriptome may not exclusively be mediated via changes in non-canonical PRC1.1 functionality, although we cannot rule out the possibility that loss of a small fraction of PRC1.1 complexes at PRC1 loci, which may be required for initiation of canonical PRC1/2-mediated repression (as was proposed by the Klose lab (Blackledge et al., 2014)) is at the heart of this loss of H2AK119ub. The slight increase in H3K27me3 was mostly seen on repressed PRC1 loci, in line with our previous notion that PRC1.1 genes are devoid of repressive H3K27me3 marks (van den Boom et al., 2016). The increase in H3K36me3 was only seen on loci bound either by PRC1.1, or by both PRC1.1 and PRC1, possibly as a consequence of loss of KDM2B demethylase activity (Figure S3). Although H3K4me3 marks remained unchanged on PRC1 or “both” loci, a slight but significant decrease was seen on non-canonical PRC1.1 loci (Figure S3). Representative screenshots for non-canonical PRC1.1 loci are shown in Figure 5D. ChIP-seq data for endogenous USP7 was generated by others in T-ALL cells (Jin et al., 2019). We overlaid those USP7 peaks with non-canonical PRC1.1 peaks and canonical PRC1 peaks that we identified in our previous study in a cohort of six primary AML CD34^+^ patient samples (van den Boom et al., 2016). This analysis revealed that 80% of PRC1.1 peaks also contained USP7, whereas only 21% of PRC1 occupied loci also contained USP7 (Figure 5E), indicating that USP7 is enriched at the majority of PRC1.1 target genes. Representative screenshots of PRC1.1 and PRC1 loci are shown in Figure 5F.

### TRIM27 and USP7 reciprocally regulate PRC1.1 stability

Our initial pull out data also revealed the presence of the E3 ligase TRIM27 in the PRC1.1 complex (Figures 1A and 1E–1H), which was previously shown to interact with USP7 (Zaman et al., 2013). To study the role of TRIM27 within PRC1.1 in more detail, we generated two independent shRNAs that efficiently downregulated TRIM27 (Figure 6A). Next, we evaluated the effect of TRIM27 knockdown on KDM2B-GFP chromatin binding and H2AK119ub levels. Downregulation of TRIM27 did not impair KDM2B chromatin binding and in fact a mild but significant increase in KDM2B chromatin occupancy at PRC1.1 target genes was noted, where H2AK119ub levels were not significantly altered (Figure 6B). Next, KDM2B-GFP pull outs were performed to investigate a potential role of TRIM27 in controlling PRC1.1 complex composition. Surprisingly, knockdown of TRIM27 resulted in loss of USP7 incorporation into PRC1.1, whereas other PRC1.1 subunits such as RING1A, RING1B, and PCGF1 still efficiently co-precipitated with KDM2B-GFP (Figure 6B). These data indicate that TRIM27 is required for the incorporation of USP7 into PRC1.1 and suggests that TRIM27-knockdown-induced loss of USP7 renders PRC1.1 insensitive to USP7 inhibition. Indeed, TRIM27 shRNA expression partially rescued the FT671-induced loss of H2AK119ub (Figure 6D). Moreover, USP7 inhibition strongly impaired cell proliferation of K562 cells, which was also partially rescued upon TRIM27 downregulation (Figure 6E).

Our finding that TRIM27 knockdown led to a loss of USP7 incorporation in PRC1.1 made us hypothesize that USP7 knockdown, in contrast to USP7 inhibition, may not affect PRC1.1 integrity. Therefore, we generated two independent USP7 shRNAs that both efficiently downregulated USP7 expression (Figure 6F). Indeed, USP7 knockdown did not affect RING1A, RING1B, and PCGF1 co-precipitation with KDM2B-GFP (Figure 6G). Importantly, TRIM27 expression was strongly reduced upon USP7 knockdown, and therefore less TRIM27 was detected in KDM2B-GFP pull outs (Figure 6G). These data highlight a reciprocal dependency between TRIM27 and USP7 for incorporation into PRC1.1. In line with these data, USP7 knockdown did not affect PRC1.1 chromatin binding and H2AK119ub deposition (Figure 6H). It has previously been reported that USP7 enzymatic activity is important to counteract TRIM27 auto-ubiquitination and subsequent proteasomal degradation (Hao et al., 2015). Because USP7-inhibitor-induced PRC1.1 depletion demands TRIM27 expression we investigated whether TRIM27 was expressed throughout our USP7 inhibition experiments. Western analysis shows that FT671 treatment did not induce a significant reduction of TRIM27 levels until 48 h after the start of the treatment, whereas P22077-induced USP7 inhibition only resulted in a mild reduction of TRIM27 levels at 48 h after treatment initiation (Figure S4A). Already within 4–24 h upon USP7 inhibition we observed loss of PRC1.1 binding and H2AK119ub marks (Figures 4D, 5, and S3). Based on these data we conclude that, within the time frame of our experiments, TRIM27 is sufficiently expressed to induce PRC1.1 destabilization. Finally, we also studied the effect of USP7 knockout in THP-1 cells utilizing a doxycycline-inducible CRISPR/Cas9 USP7 knockout model (Palazon-Riquelme et al., 2018). Cells were treated with doxycycline for 3 days and 4 days later USP7 expression was completely lost (Figure 6I). Similar to our USP7 knockdown data in K562 cells, USP7 knockout in THP-1 cells did not affect KDM2B chromatin binding and H2AK119ub levels upon USP7 deletion (Figure 6J), whereas RING1B was stably expressed (Figure S4B). Taken together, these data clearly show that both USP7 or TRIM27 knockdown do not phenocopy the effects seen upon USP7 inhibition. Our data suggest that whereas USP7 inhibition leads to PRC1.1 destabilization in the presence of TRIM27, likely as a consequence of TRIM27 E3 ligase activity, USP7/TRIM27 knockdown results in a minimal PRC1.1 complex without incorporation of the USP7/TRIM27 heterodimer (Figure 6K). To further support this hypothesis future experiments will be conducted in which the catalytic dead USP7 C223S mutant as well as wt USP7 will be reintroduced into the CRISPR-Cas9 USP7 KO lines. We anticipate that in this scenario TRIM27 would again be recruited to PRC1.1, and due to loss of de-ubiquitinase activity of the USP7 mutant this would result in a misbalance in ubiquitination of PRC1.1 proteins, resulting in disassembly of the complex, and consequently loss of H2AK119ub marks at PRC1.1 loci.

### USP7 inhibition affects PRC1.1 target gene expression

**Figure 7:**
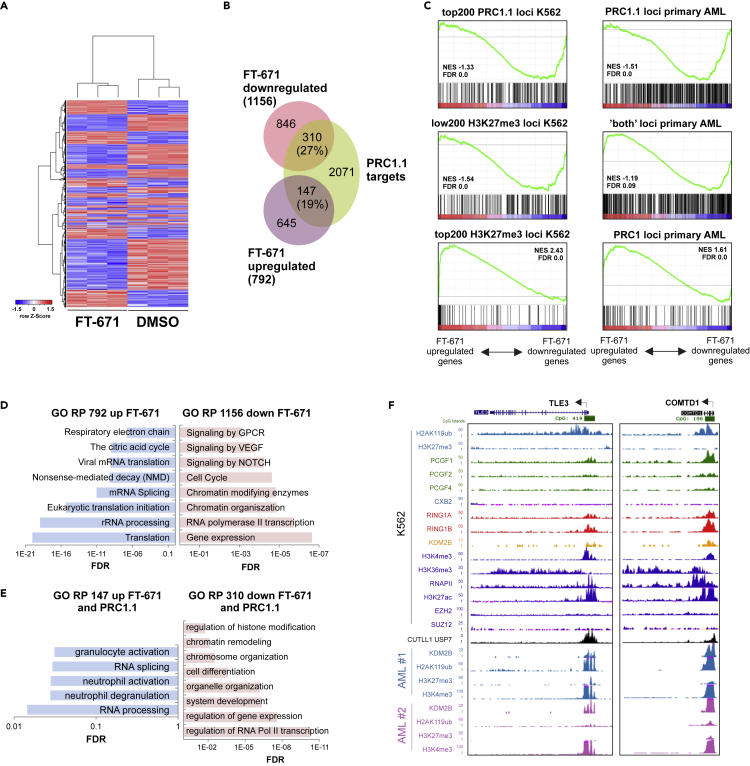
USP7 inhibition leads to transcriptional changes of PRC1.1 target genes (A) Unsupervised clustering of RNA-seq data from three independent experiments where K562 cells were treated with DMSO or FT671 (10μM, 48 h) samples. (B) Venn diagram showing overlap of significantly up- and downregulated genes (Student's t test, p < 1x10^−6^) with previously identified PRC1.1 target genes. (C) GSEA analysis showing enrichment score against ranked RNA-seq data. RNA-seq data were compared with indicated gene sets. (D) Gene ontology analyses of regulatory processes (RP) of all up- and downregulated genes in FT671-treated cells. (E) Gene ontology analyses of regulatory processes (RP) of up- and downregulated genes in FT671-treated cells that are also targeted by PRC1.1. (F) Screens shots of our ChIP-seq tracks (van den Boom et al., 2016) for H2AK119ub, H3K27me3, PCGF1, PCGF2, PCGF4, CBX2, RING1A, RING1B, and KDM2B (all GFP-fusions) in K562 cells. ChIP-seq tracks for H3K4me3, H3K36me3, RNAPII, H3K27ac, EZH2, SUZ12 (all K562), and USP7 (CUTLL1) were downloaded from ENCODE/Broad. In addition, endogenous KDM2B, H2AK119ub, H3K27me3, and H3K4me3 in two primary AML patient cell samples are shown. See also Figure S5.

Given that USP7 inhibition severely impaired PRC1.1 occupancy at several target loci we hypothesized that USP7 inhibition would also affect the expression of PRC1.1 target genes in leukemic cells. To test this, K562 cells were treated with FT671 or DMSO for 48 h, mRNA was isolated, and RNA-seq was performed. Unsupervised clustering revealed that FT671-treated samples clearly clustered away from the DMSO-treated samples (Figure 7A). We found that 1,156 genes were downregulated and 792 genes were upregulated, of which respectively 27% and 19% were overlapping with previously identified PRC1.1 target genes (van den Boom et al., 2016) (Figure 7B). Gene set enrichment studies revealed that the top 200 strongest PRC1.1 bound loci were significantly enriched for genes that were downregulated upon USP7 inhibition (Figure 7C). Downregulated genes were indeed low for the histone mark H3K27me3, whereas the top 200 H3K27me3 loci in K562 cells were in fact found to be strongly upregulated upon USP7 inhibition (Figure 7C). Very similar data were obtained when comparing our previously generated ChIP-seq data in primary AML patient samples with gene expression changes upon USP7 inhibition. Active PRC1.1 loci were significantly downregulated upon USP7 inhibition, in line with a role for PRC1.1 in inducing or maintaining and active chromatin state (Figure 7C). In contrast, canonical repressive PRC1 loci were significantly upregulated upon USP7 inhibition, whereas loci that we characterized as “both” loci, occupied by both canonical PRC1 as well as non-canonical PRC1.1 complexes, were not consistently up or downregulated (Figure 7C). Gene Ontology analyses revealed that downregulated genes upon USP7 inhibition were enriched for processes including RNApol II transcription, chromatin organization, cell cycle, and signaling by NOTCH, VEGF, and GPCR (Figure 7D). Downregulated gene sets were enriched for processes linked to translation, ribosomal RNA processing, mRNA splicing, and the respiratory electron chain (Figure 7D). Most of these GO terms were still significantly enriched when specifically focusing on PRC1.1-bound genes (Figure 7E). We also noted a consistent overlap between KDM2B peaks, and USP7 occupied loci using data that was generated previously (Jin et al., 2019; van den Boom et al., 2016) (Figure 7F).

Furthermore, RNA-seq was performed on K562 cells treated with DMSO or P22077 for 8 h, 16 h and 24 h. A number of genes were upregulated, but the largest set turned out to be downregulated upon USP7 inhibition (Figure S5A). A good overlap was seen between gene expression changes induced by P22077 and FT671 (Figure S5B). GO analysis revealed that the set of upregulated genes was enriched for GO terms such as gene expression, translation, response to stress, and apoptosis (Figure S5A). This set of upregulated genes could be consistent with a repressive function of PRC1.1, potentially in the context of repressive canonical PRC2/PRC1. The biggest transcriptional changes included genes that were downregulated upon USP7 inhibition. GO analyses revealed that this set of genes was enriched for GO terms such as metabolism, protein modification, negative regulation of RNAPII-mediated transcription, chromosome organization, and histone modifications (Figure S5C). ChIP-seq profiles are shown in Figure S5D as representative examples for PRC1.1 target genes. The tracks show clear binding of PCGF1, RING1A/1B, and KDM2B and little occupancy of canonical PRC1 proteins (PCGF2, PCGF4, CBX2). Interestingly, on the downregulated genes upon USP7 inhibition we typically did not observe any repressive H3K27me3 marks or PRC2 binding (Figure S5D), similar to what was seen with FT671, suggesting that these loci are more in an active chromatin state. These loci were also enriched for H2AK119ub and active chromatin marks H3K4me3, RNAPII, and H3K27ac. Loss of PRC1.1 binding upon USP7 inhibition was confirmed by ChIP-qPCRs for KDM2B, PCGF1, and RING1B on these four loci (Figure S5E), which coincided with reduced expression levels shown by independent quantitative RT-PCRs (Figure S5F).

### USP7 inhibition effectively targets primary AML patient cells

**Figure 8:**
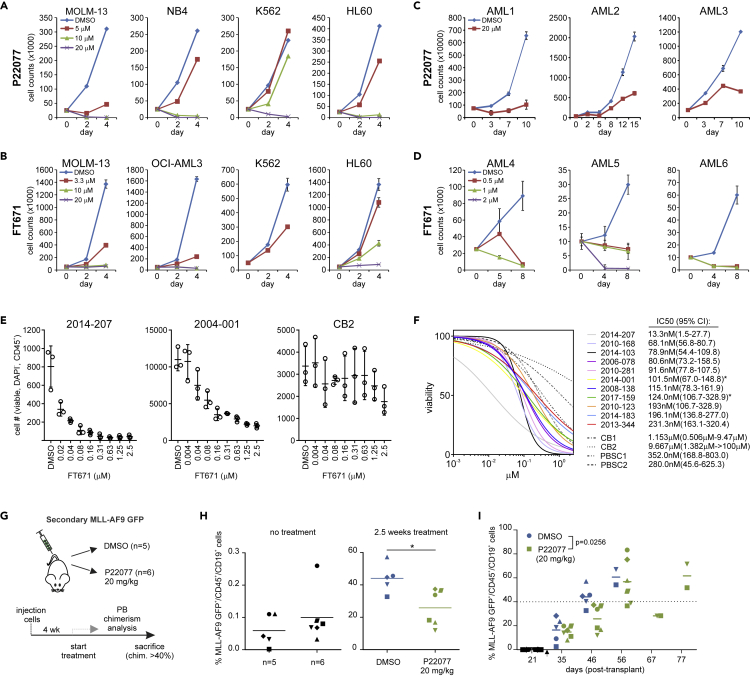
Sensitivity of AML cells toward USP7 inhibition (A) Cumulative cell growth of various AML cell lines treated with DMSO or various concentrations of P22077. (B) Cumulative cell growth of various AML cell lines treated with DMSO or various concentrations of FT671. Error bars represent SD based on measurement of biological triplicates. (C) Cumulative cell growth of primary AML patient cells (n = 3) grown on MS5 stromal cells treated with DMSO (control) or P22077. Error bars represent SD based on measurement of biological duplicates. (D) Cumulative cell growth of primary AML patient cells (n = 3) grown on MS5 stromal cells treated with DMSO or FT671. Error bars represent SD based on measurement of biological duplicates. (E) Viable cell numbers after 8 days of treatment with DMSO or increasing concentrations of FT671. Representative examples of primary AML patient cells and cord blood CD34^+^ cell samples are shown. Error bars represent SD based on measurement of biological triplicates. (F) IC50 curves and data for 11 independent primary AML samples, cord blood CD34 + cells, and mobilized peripheral blood stem cells (PBSCs), cocultured on MS5 stromal cells for 8 days in the presence of increasing amounts of FT671. Asterisk indicates TP53-mutant AMLs. (G) Experimental setup of our human CB MLL-AF9 xenograft mouse model. Here 5 x 10^4^ MLL-AF9 GFP^+^ cells from a primary leukemic mouse were IV injected into secondary recipients (n = 11). (H) Peripheral blood analysis of MLL-AF9 GFP/CD45^+^ cells, three weeks after injection prior to treatment (left) and 2.5 weeks following treatment (right). Mice were treated daily with either DMSO as control (n = 5) or 20 mg/kg P22077 (n = 6). (I) Peripheral blood chimerism levels of control and P22077 (20 mg/kg)-treated mice over the course of the experiment. Treatment was started at day 28 (4 weeks post-transplant) as indicated with an arrow. See also Figure S6.

Next, we wondered whether USP7 inhibition could be used to target AML cells. Therefore, we tested P22077 on a panel of AML cell lines and primary patient samples. USP7 inhibition severely impaired the cell growth of MOLM-13, OCI-AML3, K562, and HL60 AML cell lines (Figure 8A). Treatment of these cell lines with FT671 induced a similar reduced proliferation (Figure 8B). USP7 inhibition is known to stabilize TP53 expression, which we were able to confirm in TP53wt cell lines (Figure S6). Interestingly, however, cell lines that do not express functional TP53 (HL60 and K562), or express mutated TP53 (NB4), also showed sensitivity, indicating that, at least in those cell lines, the effect of P22077 and FT671 was independent of TP53. Given the fact that PRC1.1 functionality is essential for leukemic cell viability (van den Boom et al., 2016), we hypothesize that USP7-inhibition-induced PRC1.1 destabilization is one of the main drivers of cell death in TP53 null AML cells. Next, we assessed whether USP7 inhibition also affected the survival of primary AML cells grown in long-term stromal cocultures. Indeed, P22077 treatment induced a strong reduction in the proliferation of primary AML CD34+ cells, in three independent AML patient samples of which the first was a TP53^H179R^ mutant AML patient sample (Figure 8C). FT671 was also tested on primary AML samples, and again we observed strong reductions in proliferation (Figure 8D). Subsequently, we treated various primary AML samples, cord blood (CB) CD34^+^ cells, and CD34^+^ peripheral blood stem cells (PBSC) with an increasing dose of FT671 ranging from 4 nM to 2.5 μM for a period of 8 days on stroma. Clearly, AML samples were highly sensitive to FT671 treatment, whereas healthy CD34^+^ cells were rather insensitive (Figure 8E). IC50 curves and calculated IC50 values are displayed in Figure 7F with IC50s ranging from 13.3 nM in the most sensitive patient samples to 231.3 nM in the least sensitive patient samples. Two TP53-mutant patient samples were included (indicated with an asterix), both showing intermediate sensitivities to FT671. We also included healthy control CD34^+^ stem/progenitor cells isolated from cord blood or mobilized PB, and IC50 values were considerably higher compared to AML samples, ranging from 280.0 nM to 9.667 μM (Figure 8F).

Finally, we evaluated the effect of USP7 inhibition in our human CB MLL-AF9 xenograft mouse model (Figure 8G) (Horton et al., 2013; Sontakke et al., 2016). 5x10^4^ MLL-AF9 leukemic cells, from a primary leukemic mouse, were intravenously (IV) injected into secondary recipients (n = 11). Three weeks after injection, when 10/11 mice showed engraftment of MLL-AF9 GFP^+^/CD45^+^ cells in the peripheral blood, mice were divided into two groups that were treated either with DMSO (n = 5) or 20 mg/kg P22077 (n = 6) via intraperitoneal (IP) injections. Mice were treated daily starting four weeks post-transplant, and peripheral blood chimerism levels were monitored by regular blood sample analysis and mice were sacrificed when chimerism levels in the blood exceeded 40%. Two-and-a-half weeks after the initiation of treatment chimerism levels were significantly lower in P22077-treated mice compared with DMSO (Figure 8H). The chimerism levels for DMSO-treated mice rapidly increased to 44% (average) within 6 weeks after injection, whereas the chimerism levels of P22077-treated mice were significantly lower (25% on average). Within 60 days all control mice exceeded 40% chimerism in the blood, indicative for a full-blown leukemia and were sacrificed (Figure 8I). Bone marrow, spleen, and liver analyses showed high levels (>90%) of chimerism. Leukemia development was significantly delayed in USP7-inhibitor-treated mice, and in particular in two mice a clear response to USP7 inhibition was observed and chimerism levels remained relatively stable between day 56 and 67 post-transplant, although ultimately those mice also did develop MLL-AF9-induced leukemia after day 77 (Figure 8I).

## Discussion

Various studies have highlighted Polycomb proteins as classical regulators of stem cell self-renewal and cell lineage commitment. Also, Polycomb proteins are frequently deregulated in cancer. Therefore, a thorough understanding of Polycomb complex composition and functionalities would not only provide fundamental insight into how these epigenetic regulatory complexes control gene transcription but also might provide alternative means for targeting cancer cells. Here, we identify and functionally characterize two novel components of the non-canonical Polycomb complex PRC1.1: the deubiquitinase USP7 and E3 ubiquitin ligase TRIM27. Inhibition of USP7 enzymatic activity results in a quick disassembly of the complex, loss of PRC1.1 chromatin binding, and loss of H2AK119ub. TRIM27 and USP7 knockdown studies uncover a reciprocal dependency for their incorporation into PRC1.1. USP7 inhibition leads to robust gene expression changes, of which a significant portion is targeted by PRC1.1 in leukemic cells. Finally, we observe that primary AML patient cells are highly sensitive to USP7 inhibition, also in poor prognosis patients carrying TP53 mutations, indicating that USP7 inhibition could be an attractive alternative therapy for treatment of AML patients.

Our proteomics studies firmly establish that USP7 stably interacts with the PRC1.1 subunits KDM2B, PCGF1, and RING1B. Previous studies identified USP7 as an interactor of RING1B (Sanchez et al., 2007), and USP7 was also identified in proteomics analysis of PCGF1, RYBP, YAF2, and RING1A/B pull outs (Gao et al., 2012; Hein et al., 2015; van den Boom et al., 2016). Two other studies indicated that USP7 can also associate with the canonical PRC1 proteins BMI1/PCGF4 and MEL18/PCGF2 (Lecona et al., 2015; Maertens et al., 2010). Lecona and colleagues suggested that USP7-PRC1 interactions could be mediated via direct binding to SCML2 (Lecona et al., 2015). Indeed, although our PCGF2, PCGF4, and CBX2 (van den Boom et al., 2016) and USP7 interactome analyses (our current work) showed little or no interaction with USP7, we did find strong interactions with SCML2, suggesting that this protein might act as a bridging factor. In addition, also our stoichiometry measurements of USP7 pull outs show a strong difference between SCML2 and CBX8 stoichiometry, suggesting that the USP7-SCML2 interaction may take place outside the PRC1 complex. In line, when comparing genome-wide USP7 peaks with non-canonical PRC1.1 and canonical PRC1 peaks we observed a predominant presence of USP7 at PRC1.1 occupied loci.

The interaction of USP7 with PRC1.1 proteins is functionally relevant, because inhibition of the enzymatic activity of USP7 results in a quick disassembly of the complex and loss of chromatin binding. It is conceivable that USP7 might act on PRC1.1 by controlling the stability of individual PRC1.1 subunits by preventing polyubiquitination and consecutive proteasomal degradation. Yet, we do not have evidence that USP7 inhibition impairs PRC1.1 function by directly impacting protein stability of PRC1.1 core components such as KDM2B, PCGF1, or RING1A/B. Rather, we propose that USP7 activity might control ubiquitin levels of individual Polycomb proteins that are important for protein-protein interactions within the complex. Indeed, upon USP7 inhibition an overall increase in protein ubiquitination was observed, likely as a consequence of polyubiquitination of USP7 substrates. Although future studies will be aimed at a detailed elucidation of sites that are ubiquitinated within PRC1.1, it is clear from our data that upon USP7 inhibition KDM2B chromatin binding and protein-protein interactions within the PRC1.1 complex are lost within 24 h, whereas both endogenous as well as exogenous GFP-tagged Polycomb proteins remained stable for over 3 days. As a consequence of impaired PRC1.1 chromatin targeting, we find that H2AK119ub is lost upon USP7 inhibition. Other studies have shown that USP7 is not likely a DUB for H2AK119 (Maertens et al., 2010; Sarkari et al., 2009; van der Knaap et al., 2005), and we propose that USP7 inhibition-mediated depletion of H2AK119ub is a consequence of loss of de novo ubiquitination mediated via RING1B (Maertens et al., 2010).

USP7 was previously found to regulate UbE2E1/UbcH6, an E2 ubiquitin-conjugating enzyme, whose activity is required for PRC1-mediated H2AK119 ubiquitination (Wheaton et al., 2017). Although we cannot rule out that USP7 inhibition may affect H2AK119ub levels via this pathway, we find little interaction of USP7 with PRC1 in K562 cells and have not detected UbE2E1/UbcH6 in our USP7 pull outs. Furthermore, in contrast to observations of Wheaton and colleagues, we do not observe strongly decreased H2AK119ub levels upon USP7 knock down, suggesting the USP7-UbE2E1/UbcH6 pathway likely does not, or only mildly, contribute to the observed phenotype in leukemic cells. This is in line with previous observations by Maertens et al. where USP7 knockdown in human fibroblasts did not result in a reduction in H2AK119ub (Maertens et al., 2010).

USP7 is also known to directly bind and regulate the ubiquitination status/stability of RING1B in USP7 overexpression studies (de Bie et al., 2010; Maertens et al., 2010). In our experiments we did not observe an effect of USP7 inhibition on RING1B expression levels (Figures 3D and 3E), which may be due to the fact that we make use of pharmacological inhibition of USP7 instead of an overexpression model. Taken together, our data suggest that USP7-inhibition-induced loss of H2AK119ub is most likely caused by loss of RING1A/B activity due to PRC1.1 destabilization. Apart from a strong reduction of H2AK119ub, we observed that other chromatin marks were slightly reduced (H3K4me3) or slightly increased (H3K36me3) at PRC1.1 target genes, potentially as a consequence of loss of KDM2B H3K36-directed demethylase activity (He et al., 2008). H3K27me3 was slightly increased at PRC1 genes, potentially acting as a compensatory mechanism to prevent activation of classical Polycomb target genes.

Besides USP7, we identify another novel partner within PRC1.1, the E3 ubiquitin ligase TRIM27. USP7 and TRIM27 have previously been identified as interaction partners that can form a heterodimer in the cytoplasm, where both proteins co-regulate processes such as TNFα-induced apoptosis, endosomal protein recycling, and antiviral type I interferon signaling (Cai et al., 2018; Hao et al., 2015; Zaman et al., 2013). Mechanistically, distinct functional interactions between USP7 and TRIM27 were proposed. Zaman and colleagues showed that TRIM27 directly ubiquitinates USP7, leading to an increased USP7 DUB activity toward RIP1, resulting in a positive regulation of TNFα-induced apoptosis (Zaman et al., 2013). Hao and colleagues reported that TRIM27 (within the TRIM27/MAGE-L2 subcomplex), displays auto-ubiquitination activity, which is counteracted by USP7 DUB activity (Hao et al., 2015). Similarly, USP7-dependent deubiquitination activity toward TRIM27 was found to render TRIM27 active leading to ubiquitination/degradation of TBK1, resulting in reduced type I IFN signaling (Cai et al., 2018). We find that TRIM27 is required for incorporation of USP7 into the PRC1.1 complex. This explains why TRIM27 knockdown partially rescues USP7 inhibitor sensitivity because USP7 is simply no longer incorporated. Whether TRIM27-mediated E3 ubiquitin ligase activity is also required for USP7 incorporation into PRC1.1 remains to be determined. It will also be of interest to investigate whether other TRIM27 targets exist that are polyubiquitinated in a PRC1.1-dependent manner that are not incorporated in the complex itself but are implicated in PRC1.1-mediated transcriptional control.

Reciprocally, we find that USP7 expression is needed for incorporation of TRIM27 into the PRC1.1 complex, whereas a core PRC1.1 complex, at least including PCGF1 and RING1A/B, remains intact. We also observe that loss of USP7 leads to a reduced steady state expression of TRIM27, suggesting that TRIM27 auto-ubiquitination needs to be balanced by deubiquitinase activity of USP7, similar as in the TRIM27/MAGE-L2 complex (Hao et al., 2015), in order to prevent TRIM27 protein degradation and consequently loss of TRIM27 from PRC1.1. Remarkably, USP7 knockdown, or CRISPR-CAS9-mediated USP7 knockout, did not phenocopy USP7 enzymatic inhibition in terms of destabilization of PRC1.1 and loss of H2AK119ub levels. Apparently, in the absence of both USP7 and TRIM27, a minimal PRC1.1 complex can still exist that contains RING1A/B ubiquitin ligase activity that ensures proper H2K119 ubiquitination. These data would also argue that TRIM27 incorporation into PRC1.1 is not necessarily needed to control steady-state PRC1.1 complex activity but that it rather controls other processes at the chromatin such as transcriptional activities via its E3 ligase domain. Of note, inhibition of USP7 DUB activity also results in TRIM27 autoubiquitination and subsequent degradation, suggesting that USP7 inhibition likely mimicks a TRIM27 knockdown phenotype after prolonged treatment.

With the efficient loss of PRC1.1 binding and consequent loss of H2AK119ub from target loci upon USP7 inhibition, we were able to study PRC1.1 function in relation to gene regulation in more detail. USP7 inhibition resulted in both reduced and increased gene expression as shown by RNA-seq. A comparison with previously identified PRC1.1 bound genes revealed that the downregulated gene fraction was most enriched for PRC1.1 target genes. We hypothesize that PRC1.1 creates a transcriptionally permissive and open chromatin state, which enables transcription factors to bind and initiate gene expression. This is supported by the notion that KDM2B interacts with various complexes involved in regulating the switch from paused RNAPII to processive elongation. It will be highly interesting to further investigate the potential cross-talk between PRC1.1 proteins and transcription regulatory complexes and how this affects the epigenetic landscape and transcriptional control.

Where we previously highlighted the importance of PRC1.1 for the survival of leukemic cells using genetic studies (van den Boom et al., 2016), small molecule inhibitors would be easier to implement in a therapeutic clinical setting. USP7 is not known to be mutated in AML, but inhibition of USP7 provided an efficient means to target PRC1.1. Of course it is evident that USP7 can function in several pathways, often through regulating protein stability of tumor suppressors or epigenetic regulators (Carra et al., 2017; Felle et al., 2011; van der Horst et al., 2006) and it is particularly the TP53 pathway that is strongly controlled by USP7 (Colland et al., 2009; Fan et al., 2013; Hu et al., 2006; Ye et al., 2015). Various USP7 inhibitors have been developed, and most recently selective USP7 inhibitors were generated that destabilize USP7 substrates including MDM2 and thereby increase TP53-mediated apoptosis of cancer cells (Kategaya et al., 2017; Turnbull et al., 2017). Although we cannot exclude the possibility that interference with these pathways also contributes to the phenotype of USP7-inhibitor-treated cells, we have analyzed the TP53 status in our models and primary AML patient samples and did not observe a loss of effectiveness in TP53 mutant samples. Furthermore, although Usp7 knockout mice are embryonically lethal, deletion of Tp53 was not able to rescue this phenotype, further highlighting that TP53-independent pathways downstream of USP7 exist as well (Agathanggelou et al., 2017; Kon et al., 2010). Based on all previously identified molecular pathways that are regulated by USP7 (Kim and Sixma, 2017), our own identification of USP7 interaction partners in leukemic K562 cells, and our observation that USP7 is important for PRC1.1 functionality, we propose that the sensitivity of primary AML patient cells toward USP7 inhibition is likely a consequence of interference with USP7 functionality at various molecular levels, which includes PRC1.1 regulation but possibly also other mechanisms, given the diversity of USP7 roles that have now been identified.

In conclusion, our data reveal an important role for USP7 deubiquitinase and TRIM27 E3 ligase activity in regulating the integrity of the PRC1.1 complex. We provide insight into the recruitment of PRC1.1 to target loci and function in gene regulation and show that USP7 is a highly interesting therapeutic target in AML.

### Limitations of the study

We did not yet examine the exact sites within the PRC1.1 complex that are prone to (de)ubiquitination, which will be the focus of future studies. This will help to further unravel the molecular mechanisms via which the USP7 and TRIM27 control PRC1.1 functionality.

## STAR★Methods

### Key resources table

**Table:** 

REAGENT or RESOURCE	SOURCE	IDENTIFIER
**Antibodies**		

Anti-GFP (rabbit polyclonal)	Abcam	Cat# ab290; RRID:AB_303395
Anti-GFP (mouse monoclonal)	Santa Cruz Biotechnology	Cat# sc-9996 (B-2); RRID:AB_627695
Anti-USP7 (rabbit polyclonal)	Bethyl Laboratories	Cat# A300-033A; RRID:AB_203276
Anti-TRIM27 (mouse monoclonal)	IBL	Cat# 18791; RRID:AB_494633
Anti-RING1B (mouse monoclonal)	Abcam	Cat# ab181140; RRID:AB_2801425
Anti-STAT5 (goat polyclonal)	Santa Cruz Biotechnology	Cat# sc-835-G; RRID:AB_632447
Anti-TP53 (mouse monoclonal)	Santa Cruz Biotechnology	Cat# sc-126; RRID:AB_628082
Anti-MDM2 (rabbit polyclonal)	Santa Cruz Biotechnology	Cat# sc-813; RRID:AB_2250633
Anti-mono- and polyubiquitinated conjugates (mouse monoclonal)	Enzo Life Sciences	Cat# BML-PW8810 (FK2); RRID:AB_10541840
Anti-b-actin (mouse monoclonal)	Santa Cruz Biotechnology	Cat# sc-47778 (C4); RRID:AB_2714189
Anti-H2AK119ub (mouse monoclonal)	Cell Signalling Technology	Cat# 8240 (D27C4); RRID:AB_10891618
Anti-H3K27me3 (mouse monoclonal)	Diagenode	Cat# C15410195; RRID:AB_2753161
Anti-H3K4me3 (mouse monoclonal)	Diagenode	Cat# C15410003; RRID:AB_2616052
Anti-H3K27ac (rabbit monoclonal)	Diagenode	Cat# C15410196; RRID:AB_2637079
Anti- H3K36me3 (rabbit polyclonal)	Diagenode	Cat# C15410192; RRID:AB_2744515
IRDye 800CW goat anti-mouse IgG secondary antibody (mouse polyclonal)	LI-COR Biosciences	Cat# 926-32350; RRID:AB_2782997
Goat anti-rabbit IgG (H+L) highly cross-adsorbed secondary antibody, Alexa Fluor Plus 680 (goat polyclonal)	Thermo Fisher Scientific	Cat# A32734; RRID:AB_2633283
Goat anti-rabbit immunoglobulins/HRP (goat polyclonal)	Agilent Dako	Cat# P044801-2; RRID:AB_2617138
Rabbit anti-mouse immunoglobulins/HRP (rabbit polyclonal)	Agilent Dako	Cat# P026002-2; RRID:AB_2636929
Anti-human CD45-BV421 (mouse monoclonal)	Biolegend	Cat# 304032 (HI30); RRID:AB_2561357
Anti-human CD19-BV785 (mouse monoclonal)	Biolegend	Cat# 302240 (HIB19); RRID: AB_11218596
Anti-human CD33-APC (mouse monoclonal)	Biolegend	Cat# 303408 (WM53); RRID: AB_314351
Rabbit IgG control antibody, unconjugated	Sigma-Aldrich	Cat# I8140; RRID:AB_1163661
Anti-GFP (rabbit polyclonal)	Abcam	Cat# ab290; RRID:AB_303395

**Chemicals, peptides, and recombinant proteins**		

USP7 inhibitor P22077	Merck	Cat# 662142
USP7 inhibitor FT671	FORMA Therapeutics	(Turnbull et al., 2017)
Proteasome inhibitor MG132	Sigma	Cat# M7449

**Deposited data**		

LS-MS/MS data	This paper	PRIDE: PXD18365
RNAseq data	This paper	GEO: GSE29611

**Experimental Models: cell lines**		

K562 (AML) (Homo-sapiens)	ATCC	Cat# CCL-243
HL60 (AML) (Homo-sapiens)	ATCC	Cat# CCL-240
MV4-11 (AML) (Homo-sapiens)	ATCC	Cat# CRL-9591
MOLM13 (AML) (Homo-sapiens)	DSMZ	Cat# ACC-554
NB4 (AML) (Homo-sapiens)	DSMZ	Cat# ACC-207
OCI-AML2 (AML) (Homo-sapiens)	DSMZ	Cat# ACC-99
OCI-AML3 (AML) (Homo-sapiens)	DSMZ	Cat# ACC-582
THP1 (AML) (Homo-sapiens)	DSMZ	Cat# ACC-16
MS5 (normal, bone marrow stroma) (Mus-musculus)	DSMZ	Cat# ACC-441
Mobilized CD34+ peripheral blood samples	University Medical Center Groningen	This paper
AML CD34+ peripheral blood or bone marrow cells	University Medical Center Groningen	This paper

**Experimental models: organisms/strains**		

Mouse: NOD.Cg-Prkdcscid ll2rgtm1Wjl/SzJ	The Jackson Laboratory/ breeding at University Medical Center Groningen	JAX: 005557

**Oligonucleotides**		

shTRIM27 #1, GCTGAACTCTTGAGCCTAACC	Invitrogen Block-iT RNAi Designer	This paper
shTRIM27 #2, GGGCTGAAAGAATCAGGATTC	Invitrogen Block-iT RNAi Designer	This paper
shUSP7 #1, GCAGAGAAAGGTGTGAAATTC	Invitrogen Block-iT RNAi Designer	This paper
shUSP7 #2, GCAGAGAAAGGTGTGAAATTC	Invitrogen Block-iT RNAi Designer	This paper
TRIM27 fwd	TGGAGGGCTTCAAGGAGCAAATCC	PCR primers
TRIM27 rev	GGTTAGGCTCAAGAGTTCAGCTCGTG	PCR primers
USP7 fwd	TTGCACAGAGGCTCAACAC	PCR primers
USP7 rev	TACCTGGGCCATCCCTATAAC	PCR primers
RPL27 fwd	TCCGGACGCAAAGCTGTCATCG	PCR primers
RPL27 rev	TCTTGCCCATGGCAGCTGTCAC	PCR primers

**Recombinant DNA**		

PLKO-GFP (plasmid)	This paper	GFP version of pLKO.1-Puro

**Software and algorithms**		

MaxQuant 1.5.3.2	Cox and Mann, 2008	https://maxquant.net/maxquant/
Perseus 1.5.8.5	Tyanova et al., 2016	http://www.perseus-framework.org
David 6.8		https://david.ncifcrf.gov/home.jsp
Cytoscape 3.4		http://apps.cytoscape.org/apps/bingo
Gene Set Enrichment Analysis 2.2.2	Broad institute	https://software.broadinstitute.org/gsea/index.jsp
StrandNGS	Avadis	https://www.strand-ngs.com/
CorelDRAW Graphics Suite	CorelTM	www.coreldraw.com
GraphPad Prism 7.02	Graph Pad Prism	https://graphpad.com

**Other**		

CD34 MicroBead Kit, human	Miltenyi Biotech	Cat# 130-046-703
QIAquick PCR purification kit	Qiagen	Cat# 28106
RNeasy micro kit	Qiagen	Cat# 74004
KAPA RNA HyperPrep Kit with RiboErase (HMR	Roche	Cat# 08098131702
KAPA HyperPrep Kit	Roche	Cat# 07962363001
QuantSeq 3’ mRNA-Seq FWD Kit	Lexogen	Cat# 015.24
GFP-Trap magnetic agarose	Chromotek	Cat# gtma-20
Protein G Dynabeads	Invitrogen	Cat# 10004D
FcR blocking reagent	Miltenyi Biotech	Cat# 130-059-901
iQ SYBR Green Supermix	Bio-Rad	Cat# 170-8886

### Resource availability

#### Lead contact

Further information and requests for resources and reagents should be directed to and will be fulfilled by the lead contact, Jan Jacob Schuringa (j.j.schuringa@umcg.nl).

#### Materials availability

Plasmids and other materials generated in this study are available on request (j.j.schuringa@umcg.nl)

#### Data and code availability

All generated proteome data are available at PRIDE under PXD018365. The accession number for previously published Polycomb ChIP-seq data used for analysis in this paper is GSE54580 (van den Boom et al., 2016). Previously published ChIP-seq used for analysis include H3K4me3, H3K36me3, H3K27ac, and RNAPII/Pol2 from ENCODE/Broad Institute (GSE29611). RNA-seq data were deposited at GEO under GSE147705.

### Experimental model and subject details

#### Cell culture

The AML cell lines K562, HL60, MV4-11 (ATCC: CCL-243,CCL-240, CRL-9591), MOLM-13, NB4, OCI-AML2, OCI-AML3 (DSMZ: ACC-554, ACC-207, ACC-99, ACC-582), and THP1 containing a doxycycline-inducible CRISPR/Cas9 *USP7* knockout model (Palazon-Riquelme et al., 2018) were cultured in RPMI 1640 (BioWhittaker, Lonza, Verviers, Belgium) supplemented with 10% heat-inactivated fetal bovine serum (FCS, HyClone Laboratories, Logan, Utah, US) and 1% penicillin/streptomycin (p/s, PAA Laboratories). MS5 murine stromal cells (DSMZ: ACC-441) were cultured in alpha-MEM with 200 mM glutamine (BioWhittaker) supplemented with 10% FCS and 1% p/s. Cell lines were all tested mycoplasma free using a PCR-based assay. Primary AMLs were cultured in Gartner’s medium supplemented with G-CSF, N-plate, and IL3 (all 20 ng/ml), as described before (Sontakke et al., 2014; van Gosliga et al., 2007). CB MLL-AF9 liquid and MS5 co-cultures under myeloid or lymphoid permissive conditions were performed as described previously (Horton et al., 2013; Schuringa et al., 2004). All cultures were kept at 37°C and 5% CO2. A *USP7* knockout was induced by adding 1 μg/ml doxycycline for 3 days to THP1 cells with the CRISPR/Cas9 model. For USP7 inhibition experiments, P22077 (1-(5-((2,4-difluorophenyl)thio)-4-nitrothiophen-2-yl)ethanone) was purchased from Merck Millipore (662142) (Billerica, MA, USA). FT671 was generously provided by FORMA Therapeutics (Watertown, MA, USA) (Turnbull et al., 2017). The proteasome inhibitor MG132 was purchased from Merck Sigma-Aldrich (M7449) (Zwijndrecht, The Netherlands).

#### Patient samples

AML blasts from peripheral blood or bone marrow from untreated patients were studied after informed consent, and the protocol was approved by the Medical Ethical Committee, in accordance with the Declaration of Helsinki. Mononuclear cells were enriched by density gradient centrifugation, and CD34^+^ cells were isolated using an autoMACS (Miltenyi Biotec).

#### *In vivo* mouse models

Eight- to ten-week-old female NSG (NOD.Cg-Prkdcscid ll2rgtm1Wjl/SzJ) mice were purchased from the Centrale Dienst Proefdieren (CDP) breeding facility within the University Medical Center Groningen. Mouse experiments were performed in accordance with national and institutional guidelines, and all experiments were approved by the Institutional Animal Care and Use Committee of the University of Groningen (IACUC-RuG). Twenty-four hour prior to transplantations, mice were sub-lethally irradiated with a dose of 1.0 Gy (X-RAD 320 Unit, PXINC 2010). After irradiation mice received Neomycin (3.5 g/l) in their drinking water and soft food (RM Convalescence + BG SY (M); Special Diet Services; Witham, England) for 2 weeks. For secondary transplantations, 5 x 104 MLL-AF9 GFP cells from primary leukemic mice (CB MLL-AF9 xenograft mouse model (Horton et al., 2013; van den Boom et al., 2016) were injected IV (lateral tail vein). Peripheral blood chimerism levels were monitored by regular blood sample analysis. Mice were randomly divided into two groups, weighted and treated with DMSO as control (n=5) or 20 mg/kg P22077 (n=6) via intraperitoneal (IP) injections daily starting 4 weeks post-transplant. Prior to injections, P22077 was dissolved in DMSO (or DMSO only as control) and directly mixed with Cremophor EL (1:1). This solution was then diluted 1:4 in saline, to get an end concentration of max. 10% DMSO. Mice were humanely terminated by cervical dislocation under isoflurane anesthesia when chimerism levels in the blood exceeded 40%. Peripheral blood, bone marrow, spleen, and liver were analyzed.

### Method details

#### GFP pull outs

Pull outs were performed on nuclear extracts from K562 cells stably expressing (mutant) KDM2B-GFP, PCGF1-GFP, GFP-RING1B, or GFP-USP7. At least 80x10^6^ cells were collected for each pull out; nuclear extract preparation was done as described previously (van den Boom et al., 2013). KDM2B-GFP pull outs in SCR, TRIM27, or USP7 shRNA expressing cells were performed on 15x10^6^ cells. Pre-clearing of cell lysates was done by adding 50 μl pre-equilibrated binding control magnetic agarose beads (Chromotek) and incubated for 30 min at 4°C on a rotating platform. Then pre-cleared lysate was incubated with 70 μl pre-equilibrated GFP-Trap magnetic agarose beads (Chromotek) for 1 h at 4°C on a rotating platform. Beads were separated using a magnetic rack and six times washed in wash buffer (TBS, 0.3% IGEPAL CA-630, 1x CLAP, 0.1 mM PMSF). Bound fractions were eluted from the beads by boiling for 10 min in 2x Laemmli sample buffer.

#### In-gel trypsin digestion

Eluted fractions from GFP pull outs were loaded on a 4%–12% pre-cast NuPAGE gel (Invitrogen) and were run briefly in the gel. Gels were stained with Coomassie dye R-250 (Thermo Scientific) and subsequently destained with ultrapure water. Coomassie stained proteins were cut out in a single slice, further cut into small gel fragments, and completely destained using 70% 50 mM NH_4_HCO_3_, 30% acetonitrile (ACN). Next, gel fragments were washed in 50% 50 mM NH_4_HCO_3_, 50% ACN, 100% ACN, and subsequently dried at 55^o^C. Reduction of cysteines was performed by incubating gel fragments with 10 mM DTT dissolved in 50 mM NH_4_HCO_3_, followed by a 30 min incubation at 55°C. Next, alkylation was done by incubating the gel fragments with 55 mM iodoacetamide in 50 mM NH_4_HCO_3_ for 30 min, in the dark, at room temperature. Remaining fluid was removed, and samples were washed in 50 mM NH_4_HCO_3_ for 10 min. Then samples were washed in 100% ACN for 30 min. Fluid was removed and gel pieces were dried for 15 min at 55°C. Proteins were digested overnight at 37°C using 10 ng/μl sequencing-grade modified trypsin (Promega) diluted in 50 mM NH_4_HCO_3_. Next day, peptides were extracted using 5% formic acid followed by a second elution with 5% formic acid in 75% ACN. Samples were dried using a SpeedVac centrifuge and dissolved in 5% formic acid prior to LC-MS/MS analysis.

#### LC-MS/MS analysis

Online chromatography of the extracted tryptic peptides was performed with the Ultimate 3000 nano-HPLC system (Thermo Fisher Scientific) coupled online to a Q-Exactive-Plus mass spectrometer with a NanoFlex source (Thermo Fisher Scientific) equipped with a stainless steel emitter. Tryptic digests were loaded onto a 5 mm × 300 μm i.d. trapping micro column packed with PepMAP100 5 μm particles (Dionex) in 0.1% FA at the flow rate of 20 μL/min. After loading and washing for 3 minutes, peptides were forward-flush eluted onto a 50 cm × 75 μm i.d. nanocolumn, packed with Acclaim C18 PepMAP100 2 μm particles (Dionex). The following mobile phase gradient was delivered at the flow rate of 300 nL/min: 2%–50% of solvent B in 90 min; 50%–80% B in 1 min; 80% B during 9 min, and back to 2 % B in 1 min and held at 3% A for 19 minutes. Solvent A was 100:0 H2O/acetonitrile (v/v) with 0.1% formic acid and solvent B was 0:100 H2O/acetonitrile (v/v) with 0.1% formic acid. MS data were acquired using a data-dependent top-10 method dynamically choosing the most abundant not-yet-sequenced precursor ions from the survey scans (300–1650 Th) with a dynamic exclusion of 20 seconds. Sequencing was performed via higher energy collisional dissociation fragmentation with a target value of 2e5 ions determined with predictive automatic gain control. Isolation of precursors was performed with a window of 1.6. Survey scans were acquired at a resolution of 70,000 at m/z 200. Resolution for HCD spectra was set to 17,500 at m/z 200 with a maximum ion injection time of 110 ms. The normalized collision energy was set at 28. Furthermore, the S-lens RF level was set at 60, and the capillary temperature was set at 250^o^C. Precursor ions with single, unassigned, or six and higher charge states were excluded from fragmentation selection.

#### LC-MS/MS data analysis

Raw mass spectrometry data were analyzed using MaxQuant version 1.5.2.8 (Cox and Mann, 2008) using default settings and LFQ/iBAQ enabled, searched against the Human Uniprot/Swissprot database (downloaded June 26, 2016, 20197 entries). Further data processing was performed using Perseus software, version 1.5.6.0 (Tyanova et al., 2016). All proteome data were at PRIDE under PXD018365.

#### Data analysis

For Gene Ontology (GO) analysis we used either BiNGO (Maere et al., 2005) or DAVID Bioinformatics Resources (http://david.abcc.ncifcrf.gov/home.jsp). ChIP-seq tracks were visualized and analyzed using UCSC genome browser (http://genome.ucsc.edu). The accession number for previously published Polycomb ChIP-seq data used for analysis in this paper is GSE54580 (van den Boom et al., 2016). Previously published ChIP-seq used for analysis include H3K4me3, H3K36me3, H3K27ac, and RNAPII/Pol2 from ENCODE/Broad Institute (GSE29611).

#### Chromatin immunoprecipitation

ChIP was essentially performed as described previously (Frank et al., 2001). K562 cells stably expressing low levels of GFP-fusion vectors encoding, PCGF1-GFP, GFP-RING1B, KDM2B-GFP, or non-transduced K562 cells and CB MLL-AF9 cells were treated with DMSO, P22077, or FT671 for indicated timepoints and subsequently cross-linked. The following antibodies were used: anti-GFP (ab290, Abcam), anti-KDM2B (09-846, Merck), anti-H2AK119ub (D27C4, Cell Signaling Technology), anti-H3K4me3 (ab8580, Abcam), anti-H3K27ac (C15410196, Diagenode), and IgG (I8141, Sigma). ChIPs were analyzed by qPCR as percentage of input.

#### Chip-seq and data analysis

ChIP sequence libraries were generated using the KAPA Hyper Prep Kit (Roche Sequencing and Life Sciences) according to manufacturer’s protocol. The obtained DNA fragment libraries were sequenced on an Illumina NextSeq500 using default parameters. Obtained paired-end reads were aligned to the hg38 reference genome (Shen et al., 2014) using Burrows-Wheeler Aligner (BWA) (Li and Durbin, 2009) with default settings. Prior to alignment quality control statistics for the samples were obtained using FastQC tools. SAMtools (Li et al., 2009) was used for processing of aligned reads. Subsequently, peak calling was performed using MACS2 (Zhang et al., 2008) with the estimated fragment size and broad settings. Per histone mark peaks of separate tracks were concatenated and merged. Read counts were generated per region for every track and normalized using total read counts. To visualize the sequencing tracks in the UCSC browser or the Integrative Genomics Viewer (Robinson et al., 2011) the total number of overlapping fragments at each position in the genome were determined using BEDtools. BedGraph files were converted to BigWig files using UCSC bedGraphToBigWig. For the generation of average plots and heatmaps ngs.plot (Shen et al., 2014) was used. Using ngs.plot the number of reads for each gene were counted and normalized for the total number of mapped reads per sample. Average plots were generated on the region -/+ 5 Kb around the TSS or gene body. Scatterplots showing the correlation of ChIP-seq read counts of cells treated with DMSO or FT671 were generated by selecting the maximum read values on the region −/+ 5kb around the TSS or gene body (H3K36me3) per gene. Maximum values per gene on the region −/+ 5kb around the gene body were also selected for boxplots showing the ChIP level distributions of each histone mark on previously selected PRC1.1, PRC1, and both PRC1.1 and PRC1 targets. Data were deposited at GEO under GSE147705.

#### Generation of constructs

Generation of lentiviral pRRL SFFV PCGF1-GFP, pRRL SFFV PCGF2-GFP, pRRL SFFV PCGF4-GFP, pRRL SFFV GFP-CBX2, and pRRL SFFV GFP-RING1B vectors was described previously (van den Boom et al., 2016). To generate the pRRL SFFV KDM2B-GFP vector, *KDM2B* was PCR amplified from cDNA in two parts, fragment 1 (startcodon-RsrII) and fragment 2 (RsrII-second-last codon). Both fragments were independently subcloned into pJet1.2, resulting in pJet1.2 *KDM2B* fragment 1 and pJet1.2 *KDM2B* fragment 2, which were subsequently verified by sequencing. Next, pJet1.2 *KDM2B* fragment 2 was digested using RsrII and XbaI digestion and ligated into pJet1.2 *KDM2B* fragment 1 also digested with RsrII and XbaI, resulting in a pJet1.2 KDM2B construct lacking the stop codon. Finally, the *KDM2B* ORF was subcloned into the pRRL SFFV GFP vector using AgeI, resulting in the pRRL SFFV KDM2B-GFP construct. To generate the pRRL KDM2B short form (SF)-GFP lentiviral vector we PCR amplified the region stretching from the the short form startcodon until the RsrII site. After subcloning into pJet1.2, and sequence verification, this fragment was subcloned into the pRRL SFFV KDM2B-GFP vector using EcoRI/RsrII digestion, replacing the 5’ side of the KDM2B long form. All other KDM2B mutants were generated by inverted PCR on the pJet1.2 KDM2B construct, followed by subcloning into the pRRL SFFV GFP vector. For generation of the lentiviral pRRL SFFV GFP-TEV-FLAG-3C-USP7 vector we digested pcDNA5.1_FRT-TO_PURO_(N)GFP-TEV-FLAG-3C-USP7 with HindIII and XhoI (both Klenow blunted) and ligated the fragment into pRRL SFFV GFP-RING1A cut with BamHI and MluI (both Klenow blunted). A lentiviral pRRL SFFV-His-Ubiquitin-mBlueberry2 vector was generated by PCR amplification of His-Ubiquitin from pCl His-Ubi (Addgene) plasmid using primers including BamHI sites, followed by ligation into pJet1.2 and sequence verification. Subsequently, His-Ubiquitin subcloned into pRRL SFFV-IRES-mBlueberry2 using BamHI digestion. Generation of lentiviruses and subsequent transductions were performed as described previously (van den Boom et al., 2016).

#### Flow cytometry analysis

Flow cytometry analyses were performed on a BD LSR II (Becton Dickinson (BD) Biosciences), and data were analyzed using FlowJo (Tree Star Inc, Ashland, OR, USA). Cells were sorted on a MoFlo XDP or MoFLo Astrios (Beckman Coulter). *In vivo* engraftment levels were analyzed in peripheral blood (PB), bone marrow, liver, and spleen. Prior to staining, cells were blocked with anti-human FcR block (MACS miltenyi Biotec) and anti-mouse CD16/CD32 block (BD Biosciences) and stained with CD45-BV421 (HI30, Biolegend), CD19-BV785 (HIB19, Biolegend), and CD33-APC (WM53, Biolegend) at 4°C for 30 min.

#### Isolation ubiquitinated proteins

For detecting His-tagged ubiquitinated proteins cells were lysed in 1 ml denaturing lysis buffer (6M Guanidium-HCl, 100 mM Na_2_HPO_4_ x 2H_2_O, 10 mM Tris-HCl pH 8.0, 5 mM Imidazole, 10 mM β-mercaptoethanol) and sonicated on ice. Lysates were pre-cleared by centrifugation at 14000 rpm at 4°C, and five volumes of denaturing lysis buffer was added. His-tagged ubiquitinated proteins were purified by adding 75 μl pre-equilibrated Ni-NTA magnetic agarose beads (Jena Bioscience) and incubated for 4 h on a rotating wheel. Beads were separated using a magnetic rack and washed 1x in denaturing lysis buffer without Imidazole, 1x in wash buffer pH 8.0 (8M Urea, 100 mM Na_2_HPO_4_ x 2H_2_O, 10 mM Tris-HCl pH 8.0, and 10 mM β-mercaptoethanol), 1x in wash buffer pH 6.3 (8M Urea, 100 mM Na_2_HPO_4_ x 2H_2_O, 10 mM Tris-HCl pH 6.3, 10 mM β-mercaptoethanol) plus 0.2% Triton X-100, and 1x in wash buffer pH 6.3 plus 0.1% Triton X-100. His-tagged ubiquitinated proteins were eluted from the beads by adding 75 μl elution buffer (200 mM Imidazole, 150 mM Tris-HCl pH 6.7, 30% glycerol, 5% SDS and 720 mM β-mercaptoethanol) and incubated for 20 min. on a rotating wheel. Elution samples were diluted 2x in Laemmli sample buffer containing 10% β-mercaptoethanol and immediately boiled for 5 min prior to western blot analysis.

#### Western blotting

Western blotting was performed as previously described (van den Boom et al., 2013). The following primary antibodies were used in this study: anti-KDM2B (09-864, Merck), anti-USP7 (A300-033A, Bethyl Laboratories), anti-TRIM27 (18791, IBL), anti-RING1A (ab180170, Abcam), anti-RING1B (ab181140, Abcam), anti-PCGF1 (ab183499, Abcam), anti-STAT5 (sc-835-G, Santa Cruz Biotechnology), anti-TP53 (sc-216, Santa Cruz Biotechnology), anti-MDM2 (sc-813, Santa Cruz Biotechnology), anti-GFP (sc-9996, Santa Cruz Biotechnology), anti-GFP (ab290, Abcam), anti-Ubiquitin (FK2, Enzo Life Sciences), anti-H2AK119ub (D27C4, Cell Signaling), and anti-b-Actin (C4, Santa Cruz Biotechnology). Secondary antibodies used included either goat anti-mouse IRDye 800 or goat anti-rabbit IgG (H+L) Alexa Fluor 680 (Invitrogen) for imaging using an Odyssey CLx Imaging System (Li-Cor Biosciences), or goat anti-rabbit immunoglobulins/HRP (Agilent-Dako), and rabbit anti-mouse immunoglobulins/HRP (Agilent-Dako) for imaging using a ChemiDoc XRS+ System (Biorad).

#### RNA-seq analysis and quantitative real-time PCR

RNA samples for sequencing were prepared for K562 cells treated with DMSO and FT671 (10 μM) for 48 h and K562 cells treated with DMSO and P22077 (30 μM) for 4 h, 8 h, 16 h, and 24 h. Total RNA was isolated using the RNeasy Mini Kit from Qiagen (Venlo, The Netherlands) according to the manufacturer’s recommendations. Initial quality check and RNA quantification of the samples were performed by automated gel electrophoresis on the 2200 TapeStation System (Agilent Technologies). Sequence libraries were generated using the KAPA RNA HyperPrep kit with riboErase (HMR) (Roche Sequencing and Life Sciences) according to manufacturer’s protocol (DMSO/FT671 samples) or the Lexogen Quantseq 3’ prep kit (Lexogen GmbH) according to the manufacturer’s recommendations (DMSO/P22077 samples). The obtained cDNA fragment libraries were sequenced on an Illumina NextSeq500 using default parameters. Obtained single-end reads of P22077 treated cells were analyzed using the Strand Avadis NGS (v3.0) software (Strand Life Sciences Pvt.Ltd). Quality trimmed reads were aligned to the hg19 reference genome. For normalization and to find differentially expressed genes the DESeq package (Anders and Huber, 2010) was used. Obtained paired-end reads of FT671-treated cells were aligned to the hg38 reference genome using STAR aligner (Dobin et al., 2013) in two-pass mode with default parameters. Simultaneously, read counts were collected, which were used as input for the DESeq2 package (Love et al., 2014) to find differentially expressed genes. Genes with an adjusted p-value < 0.00001 were found to be differentially expressed and were used in further analysis. Differentially expressed genes were clustered using hierarchical clustering with Euclidean distances (stats package in R). Data were deposited at GEO under GSE147705.

For quantitative RT-PCR, RNA was reverse transcribed using the iScript cDNA synthesis kit (Bio-Rad) and amplified using SsoAdvanced SYBR Green Supermix (Bio-Rad) on a CFX384 Touch Real-Time PCR Detection System (Bio-Rad). RPL27 was used as housekeeping gene. Primer sequences are listed in the key resources table.

### Quantification and statistical analysis

Figure 1E: numbers indicate the average total spectra counts, as measured in triplicate, corrected for maximally identifiable peptides based on in silico protein digests.

Figures 1F, 2C, 2D, and 3B: pullouts were performed in triplicate, and LC-MS/MS data were analyzed using MaxQuant and Perseus software. Statistical analysis was performed using Student’s t test (false discovery rate (FDR) <0.01; fold change (FC) >10).

Figures 2E and 3C: intensity-based absolute-quantification (iBAQ)-value-based calculation of relative stoichiometry values relative to BCOR.

Figures 4A–4E, and 6A: error bars represent SD of technical qPCR replicates.

Figures 4F, 8B, and 8E: error bars represent SD of three biological replicate experiments.

Figures 6B, 6D, 6H, and 6J: error bars representing SD based on three independent experiments; statistical analysis was performed using Student’s t test; ∗p<0.05 and ∗∗p<0.01.

Figures 6E, 8C, and 8D: error bars represent SD based on two independent experiments.

Figure 7B: Venn diagram showing overlap of significantly up- and downregulated genes (Student’s t test; p<1x10^−6^) with previously identified PRC1.1 target genes.

Figure 8F: IC50 curves were calculated using GraphPad Prism 7.02.

Figure 8H: Student’s t test; ∗p<0.05.

## Supplemental information

Document S1. Figures S1–S6

Table S1. Mass spec data KDM2B-GFP pull out, related to Figure 1

Table S2. Spectral counts (mutant) KDM2B pull outs, related to Figure 1

Table S3. Comparison mass spec data KDM2B LF-GFP and KDM2B-d(LRR)-GFP pull outs, related to Figure 1

Table S4. Mass spec data GFP and GFP-USP7 pull outs, related to Figure 2

Table S5. Mass spec data from GFP-RING1B and PCGF1-GFP pull outs in DMSO/P22077-treated cells, related to Figure 3

Table S6. Oligonucleotides, related to Figures 4 and 6

Document S2. Article plus supplemental information
