# Supplementary material for: The USP7-TRIM27 axis mediates non-canonical PRC1.1 function and is a druggable target in leukemia
Source: iScience. 2021 Apr 16;24(5):102435. doi: 10.1016/j.isci.2021.102435 (PMC8169803; doi:10.1016/j.isci.2021.102435)
Supplement: Document S1. Figures S1–S6 [file mmc1.pdf]

iScience, Volume *24*

Supplemental information

The USP7-TRIM27 axis mediates non-canonical PRC1.1 function and is a druggable target in leukemia

Henny Maat, Tjerk Jan Atsma, Shanna M. Hogeling, Aida Rodríguez López, Jennifer Jaques, Mirjam Olthuis, Marcel P. de Vries, Chantal Gravesteijn, Annet Z. Brouwers-Vos, Nisha van der Meer, Suzan Datema, Jonas Salzbrunn, Gerwin Huls, Roy Baas, Joost H.A. Martens, Vincent van den Boom, and Jan Jacob Schuringa

Supplemental Figures

The USP7-TRIM27 axis as part of non-canonical PRC1.1 is a druggable target in leukemia

Henny Maat[1,6], Tjerk Jan Atsma[1,6], Shanna M. Hogeling[1], Aida Rodríguez López[1], Jennifer Jaques[1], Mirjam Olthuis[1], Marcel P. de Vries[2,3], Chantal Gravesteijn[1], Annet Z. Brouwers-Vos[1], Nisha van der Meer[1], Suzan Datema[1], Jonas Salzbrunn[1], Gerwin Huls[1], Roy Baas[4], Joost H.A. Martens[5], Vincent van den Boom[1,*] and Jan Jacob Schuringa[1,7,*]

Figure S1

A

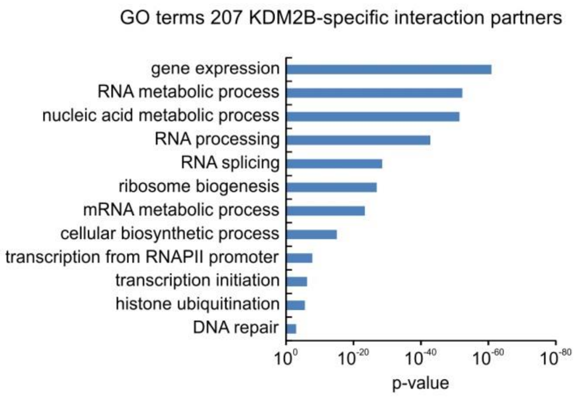

B

Gene ID	control	LF	SF	Δ(CxxC)	Δ(PHD)	Δ(FBOX)	Δ(LRR)	
**CCNT1**	0	17	23	1	20	17	14	pTEFb
**CDK9**	0	31	33	0	19	33	24	
**MLLT1/ENL**	0	76	58	21	64	106	38	MLL-fusion partners/ELL ass. proteins
**AFF1**	0	41	21	0	40	81	30	
**AFF4**	0	59	21	0	53	107	38	
**ELL**	0	30	0	0	17	50	10	
**EAF1**	0	8	0	0	8	25	0	
**SUPT16H**	0	69	123	21	158	95	43	FACT
**SSRP1**	0	113	168	41	207	128	72	
**PAF1**	0	84	39	1	187	100	32	PAF
**CDC73**	0	74	54	5	158	75	30	
**CTR9**	0	56	27	0	156	65	28	
**LEO1**	0	30	17	0	92	54	18	
**WDR61**	0	49	24	0	120	58	7	
**MED1**	0	24	3	29	7	0	0	Mediator
**MED12**	0	28	18	32	17	0	0	
**MED13**	0	2	0	6	0	0	0	
**MED13L**	0	7	0	6	0	0	0	
**MED14**	0	15	4	27	11	0	0	
**MED15**	0	14	0	17	3	0	0	
**MED16**	0	9	0	8	0	0	0	
**MED17**	0	8	0	12	2	0	0	
**MED23**	0	11	4	15	6	0	0	
**MED24**	0	8	2	10	3	0	0	

#spectra corrected for max peptides

Figure S1. KDM2B interacts with transcription regulatory complexes. Related to Figure 1. (**A**) Gene Ontology analysis of 207 KDM2B-specific interaction partners. (**B**) LC-MS/MS data of (mutant) KDM2B-GFP pull outs from K562 cells expressing the long (LF) or short (SF) isoform of KDM2B, or KDM2B-GFP lacking either the CxxC domain, PHD domain, FBOX or LRR region, highlighting co-precipitating transcription regulatory complexes.

Figure S2

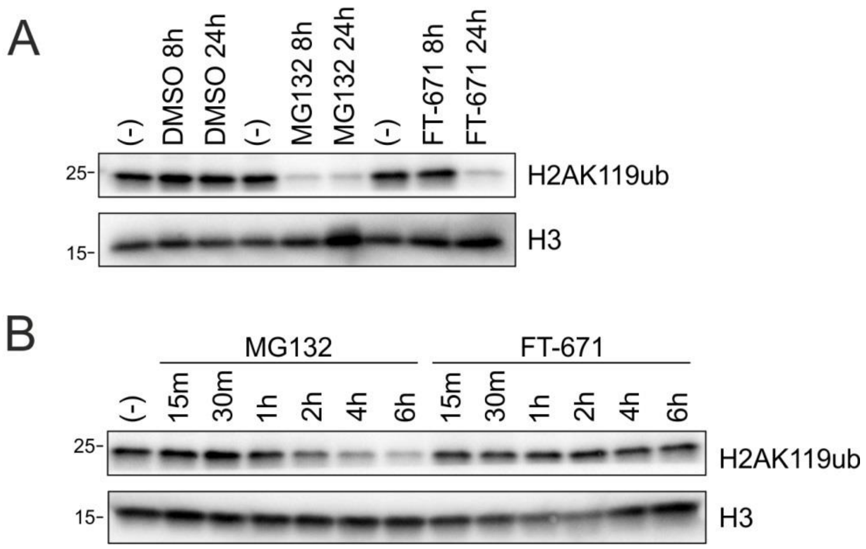

Figure S2. Kinetics of H2AK119ub loss upon proteasome inhibition or USP7 inhibition. Related to Figure 4. K562 cells were treated with DMSO as controls, MG132 (10 μM) or FT671 (10 μM) for 8-24 hrs (**A**) or 15 min to 6 hrs (**B**) and cell lysates were used for Western blotting using antibodies agaionst H2AK119ub or H3.

Figure S3

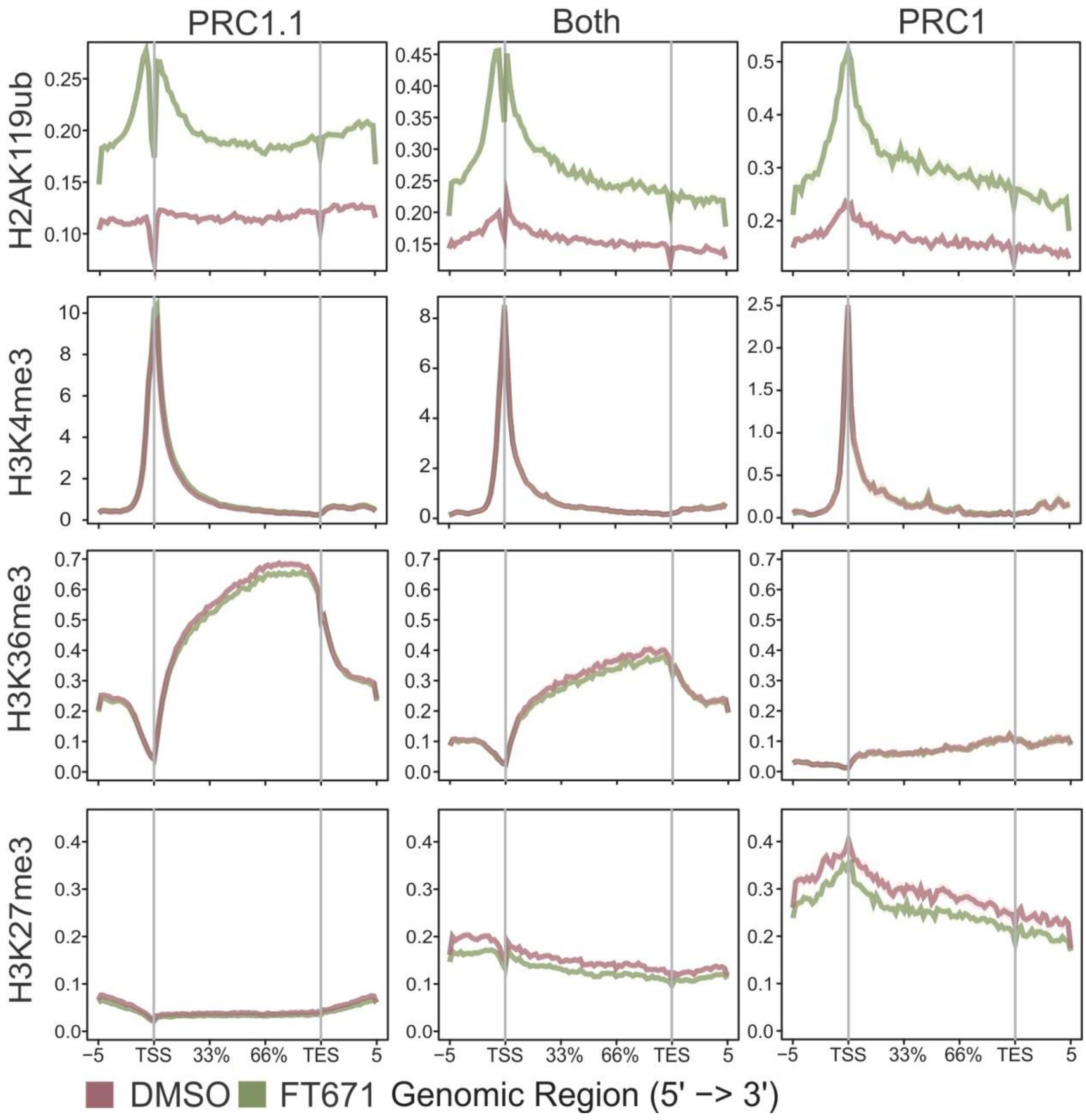

Figure S3. Genome-wide loss of H2AK119ub marks upon USP7 inhibition. Related to Figure 5. ChIP-seq on K562 cells treated with either DMSO or FT671 (24 h, 10 µM) using antibodies against H2AK119ub, H3K4me3, H3K36me3 or H3K27me3 for PRC1.1, PRC1, or loci occupied by both.

Figure S4

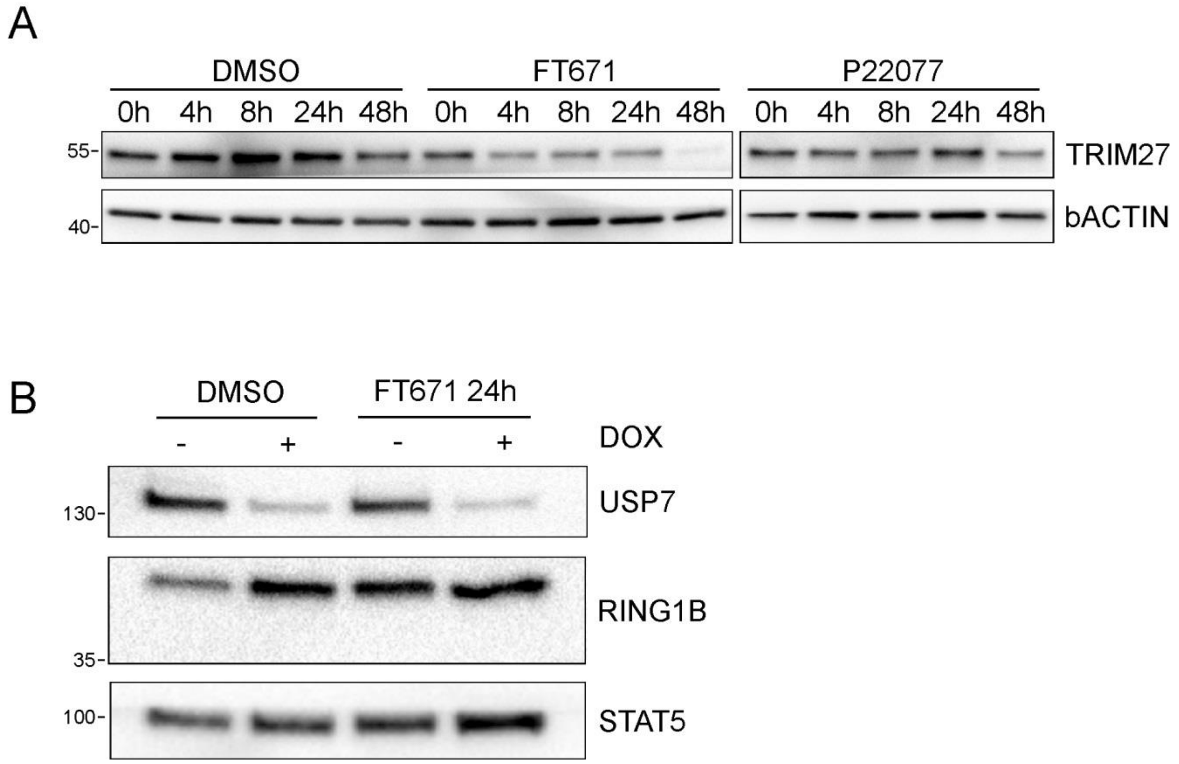

Figure S4. TRIM27 and RING1B expression levels upon USP7 inhibition or inducible USP7 deletion. Related to Figure 6. (**A**) Western analysis of K562 cells treated with DMSO, FT671 (10 μM), or P22077 (30 μM) for indicated time points and stained with antibodies directed against TRIM27 and bACTIN. (**B**) Western analysis of doxycyclin-inducible USP7 knockout cells (THP1) stained with antibodies directed against USP7, RING1B, and STAT5.

Figure S5

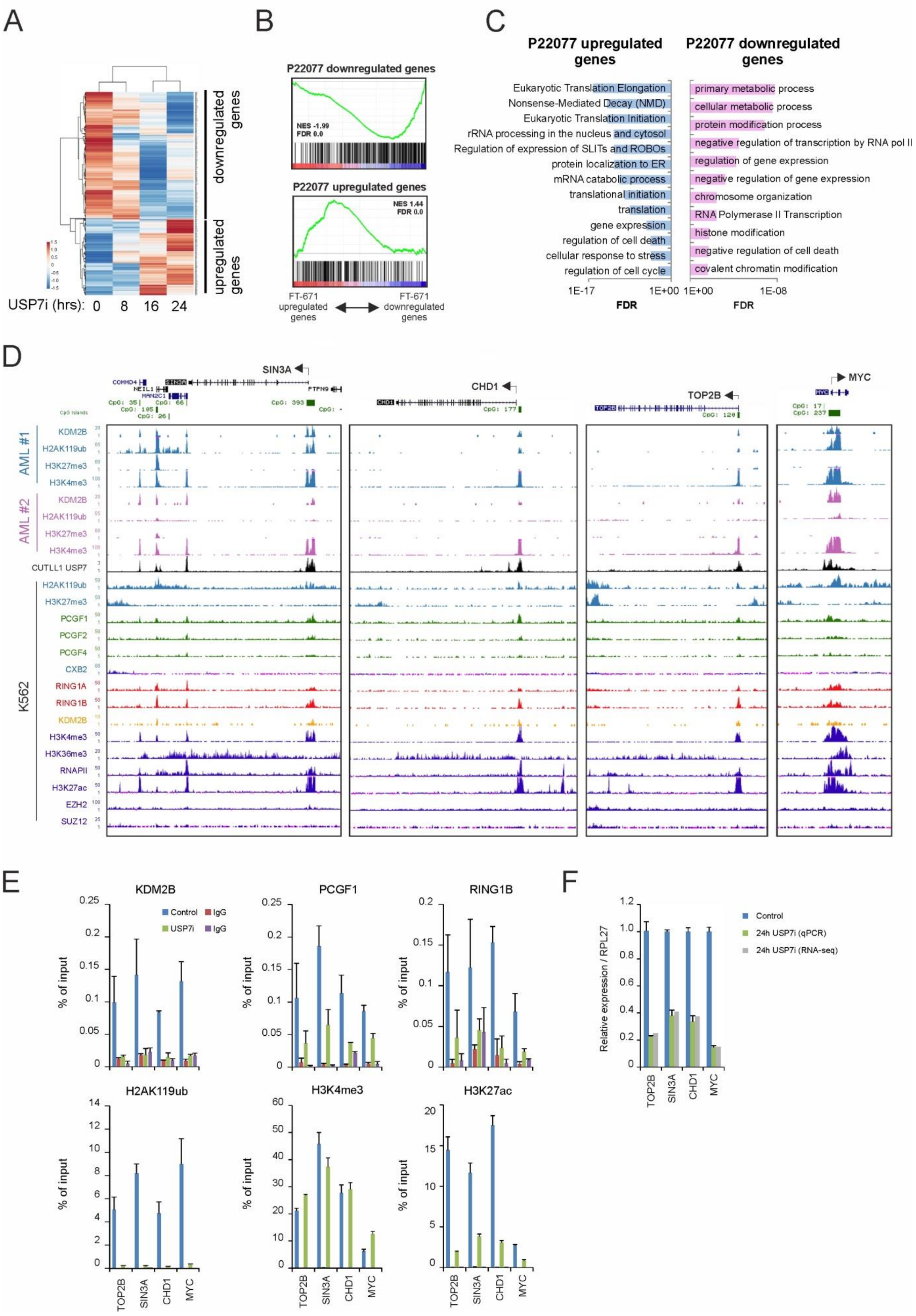

A
downregulated genes
upregulated genes
USP7i (hrs): 0 8 16 24
B
P22077 downregulated genes
NES -1.99
FDR 0.0
P22077 upregulated genes
NES 1.44
FDR 0.0
FT-671 upregulated genes
FT-671 downregulated genes
C
P22077 upregulated genes
Eukaryotic Translation Elongation
Nonsense-Mediated Decay (NMD)
Eukaryotic Translation Initiation
rRNA processing in the nucleus and cytosol
Regulation of expression of SLITs and ROBOs
protein localization to ER
mRNA catabolic process
translational initiation
translation
gene expression
regulation of cell death
cellular response to stress
regulation of cell cycle
1E-17 1E+00
FDR
P22077 downregulated genes
primary metabolic process
cellular metabolic process
protein modification process
negative regulation of transcription by RNA pol II
regulation of gene expression
negative regulation of gene expression
chromosome organization
RNA Polymerase II Transcription
histone modification
negative regulation of cell death
covalent chromatin modification
1E+00 1E-08
FDR
D
SIN3A
CHD1
TOP2B
MYC
AML #1
AML #2
K562
KDM2B
H2AK119ub
H3K27me3
H3K4me3
CUTLL1 USP7
PCGF1
PCGF2
PCGF4
CXB2
RING1A
RING1B
H3K36me3
RNAPII
H3K27ac
EZH2
SUZ12
E
KDM2B
PCGF1
RING1B
H2AK119ub
H3K4me3
H3K27ac
Control
IgG
USP7i
% of input
TOP2B
SIN3A
CHD1
MYC
F
Relative expression / RPL27
Control
24h USP7i (qPCR)
24h USP7i (RNA-seq)
TOP2B
SIN3A
CHD1
MYC

Figure S5. P22077-mediated USP7 inhibition results in gene expression changes similar to FT671 treatment. Related to Figure 7. (**A**) Supervised clustering of RNA-seq data from K562 cells treated with DMSO or 30μM P22077 for 8, 16, or 24 hrs. (**B**) GSEA analysis of ranked FT671-induced transcriptional changes compared to genes up- or down-regulated by P2207 treatment. (**C**) Gene ontology analysis of P22077 up- and down-regulated genes showing enriched biological processes. (**D**) Screen shots of various ChIP-seq tracks of PRC1.1 subunits and histone marks at genes that were downregulated upon P22077 treatment. (**E**) ChIP-qPCRs of endogenous KDM2B, PCGF1-GFP, GFP-RING1B and H2AK119ub in control or P22077 (30μM, 72h) treated K562 cells, and H3K4me3 and H3K27ac in cells treated with P22077 (30μM) for 16 hrs. Error bars represent SD of technical qPCR replicates. (**F**) Validation of relative gene expression levels of PRC1.1 target genes by qRT-PCR in control and P22077 treated cells (24h). Error bars represent SD of technical qPCR replicates.

Figure S6

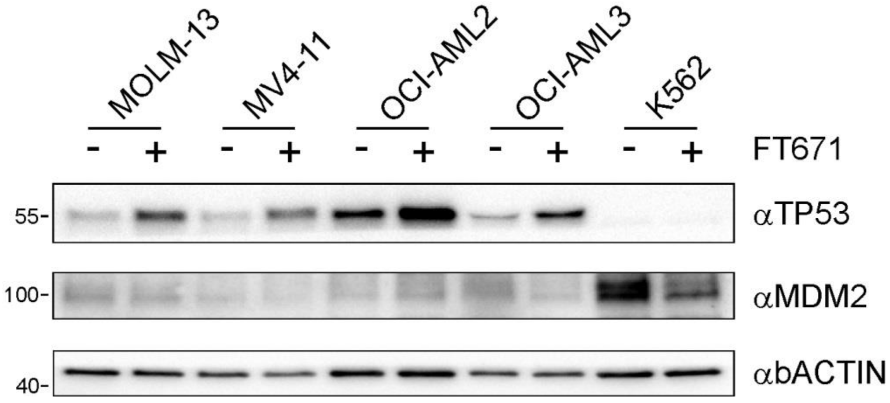

Figure S6. Analysis of TP53 and MDM2 protein expression upon USP7 inhibition in AML cell lines. Related to Figure 8. Western analysis of lysates from various AML cell lines treated with DMSO or 10 μM FT671 for 24 hours. Blots were probed with antibodies directed against TP53, MDM2, and bACTIN.
